# Proteomic Changes in the Cytoplasmatic Fraction of Weaned Piglets’ Liver and Kidney Under Antioxidant and Mycotoxin Diets

**DOI:** 10.3390/antiox14101216

**Published:** 2025-10-09

**Authors:** Roua Gabriela Popescu, Anca Dinischiotu, Andreea-Angelica Stroe, Sergiu Emil Georgescu, George Cătălin Marinescu

**Affiliations:** 1 Independent Research Association, 012416 Bucharest, Romania; roua@independent-research.ro; 2 Blue Screen SRL, 012416 Bucharest, Romania; 3 Department of Biochemistry and Molecular Biology, Faculty of Biology, University of Bucharest, Splaiul Independenței 91–95, 050095 Bucharest, Romania; anca.dinischiotu@bio.unibuc.ro (A.D.); angelica-andreea.stroe@bio.unibuc.ro (A.-A.S.); sergiu.georgescu@bio.unibuc.ro (S.E.G.)

**Keywords:** piglets, mycotoxins, proteomics, DIA, feed additives, antioxidant effect, antioxidants proteomics, cytoplasmatic fraction, pathway analysis

## Abstract

Mycotoxin contamination represents a major risk to both human and animal health. Antioxidants can mitigate some of these effects through free radical scavenging, reduction in oxidative stress, and anti-inflammatory and immunomodulatory actions. This work investigated the potential of antioxidants derived from grapeseed and sea buckthorn to mitigate the adverse effects of aflatoxin B1 (AFB1) and ochratoxin A (OTA) in weaned piglets. An unbiased Data-Independent Acquisition (DIA) proteomic approach was used to analyse the impact of OTA- and AFB1-contaminated diets on liver and kidney cytoplasmic metabolism, particularly focusing on the conjugation phase. Our results indicate that several toxic effects of these mycotoxins were partially alleviated by dietary antioxidant supplementation. Additionally, in kidneys, some of the effects are synergistically amplified, such as proteins involved in fatty acid degradation, peroxisome, PPAR signalling, translation, the TCA cycle, and excretion pathways. Inclusion of antioxidants in the animal diet can have beneficial effects. Nevertheless, caution is advised; synergistic effects can occur with potentially more serious consequences than the effects of mycotoxins alone.

## 1. Introduction

Certain strains of fungi growing on food and feed crops produce small and stable toxic secondary metabolites known as mycotoxins, which occur in various stages of food production [1,2]. Mycotoxin contamination represents a threat for human and animal health, especially through the negative effects on hormonal, immune, and DNA repair functions as well as lipid, carbohydrate, and amino acid metabolism [3,4]. Mycotoxins enter the body through consumption of contaminated processed food or its primary constituents, such as cereals, oilseeds, fruits, and vegetables, either directly or as byproducts [5,6]. In addition, humans are more exposed to mycotoxin contamination through the consumption of milk, eggs, meat, and fish derived from animals that were fed a mycotoxin-contaminated diet [7,8]. Following absorption through the digestive system, mycotoxins are distributed in different organs of the body and metabolised, mainly by the liver and kidney [9].

Once uptaken in the body, mycotoxins are metabolised by the biotransformation of the xenobiotic pathway. In phase I, namely the modification phase, members of the cytochrome P450 family catalyse mainly the oxygenation, dealkylation, and epoxidation reactions [10]. In phase II or the conjugation phase, glutathione S-transferases, sulfotransferases, N-acetyltransferases, and uridine 5′-diphospho-(UDP)-glucuronosyltransferases are involved [11]. Finally, in phase III, respectively, the excretion phase, transporters present in the liver, kidneys, and intestines facilitate the elimination of metabolites formed in cells in an active manner [12,13].

Currently, numerous strategies have been developed to prevent, reduce, or even eliminate mycotoxin contamination in animal feed through biological, chemical, and physical detoxification methods, some of them allowing the degradation of mycotoxins and their corresponding metabolites, and maintain the nutritional value of food products, without introducing other potentially toxic substances into biological systems [14,15,16]. Among these, the most promising method is the addition of antioxidants derived from plants to feed to reduce the harmful effects caused by the presence of mycotoxins on animal health. Several studies suggest that antioxidants can counteract chemical carcinogenesis by being administered before or concurrently with the carcinogen, thereby supporting their inhibitory potential against the effects of mycotoxins [17,18,19]. Although adding antioxidants to feed could support the circular economy [20], it is usually not feasible due to various factors. In addition, not all antioxidants are suitable for all types of feed due to problems of colour, taste, solubility, stability, biological activity, and interaction with other feed components (like proteins or phenolic compounds) [21].

An interesting fact is that in certain cases, the sources of potential protective agents are also a source of mycotoxins, especially the most common AFB1 and OTA. This scenario can be encountered with coffee, tea, citrus, and herbs [22,23]. Therefore, the prevention of effects should strictly promote anti-mycotoxin action. To date, most studies have tried to confirm the beneficial effects of adding food byproducts rich in antioxidants to the animal diet. However, in recent years, with the development of proteomics and metabolomics methods, new studies have highlighted their side effects or even negative effects [24,25]. Antioxidants are believed to counteract some of the effects of mycotoxins due to their ability to scavenge free radicals, reduce oxidative stress, and have anti-inflammatory and immunomodulatory properties [26]. The specific mechanisms by which antioxidants mitigate or counteract the effects of mycotoxins may vary depending on the type of antioxidant and the mycotoxin involved. For example, grapeseed contains polyphenols and proanthocyanidins that help to neutralise the free radicals, prevent lipid peroxidation, and support detoxifying enzymes [27,28], while sea buckthorn is rich in carotenoids, flavonoids, and vitamins C and E, which enhance the antioxidant defences, stabilise cell membranes, and modulate immune responses [29,30]. These mechanisms are especially relevant against AFB1 and OTA, which induce oxidative stress and disrupt the detoxification pathway [31,32].

In our previous investigation about the effects of feed mycotoxin contamination with AFB1 (62 μg/kg) and OTA (479 μg/kg), and the potential of the addition of a byproduct mixture of antioxidants to mitigate these effects on the microsomal fraction of weaned piglets’ liver, we found that some protein expressions were affected by antioxidants [25]. However, these observations only apply to the microsomal fraction that contains mostly proteins involved in phase I biotransformation, while phase II or conjugation enzymes are located within the cytoplasmatic fraction.

In this study, we aimed to investigate the proteomic level impact of the OTA- and AFB1-contaminated diet in piglets using an unbiased Data-Independent Acquisition (DIA) approach, showing how the addition of grapeseed and sea buckthorn meal to the diet impacts liver and kidney cytoplasmatic metabolism in the conjugation phase.

## 2. Materials and Methods

### 2.1. Animal Experiments

For a 30-day feeding trial, a total of 40 weaned piglets of the TOPIGS-40 hybrid breed (average body weight of 9.11 kg ± 0.03 kg) were divided into four groups. The control group (C) was fed with a standard diet for post-weaning piglets. Experimental groups included the following: antioxidant group (A), which received the basal diet supplemented with a 5% mixture of grapeseed and sea buckthorn meal byproducts; mycotoxin group (M), which received the basal diet contaminated with 62 μg/kg of aflatoxin B1 (AFB1) and 479 μg/kg of ochratoxin A (OTA); and antioxidant plus mycotoxin co-administration group (AM), which received the basal diet with a byproduct mixture and contaminated with AFB1 and OTA. Diets were formulated according to Popescu et al. [33], where the grapeseed and sea buckthorn byproducts used as dietary supplements were characterised for their macronutrient content, fatty acid profile, polyphenol and flavonoid composition, and mineral content, providing essential information on their antioxidant, anti-inflammatory, and nutritional properties. Animal performance and biomarkers of liver and kidney function have been previously reported [34]. All animals had *ad libitum* access to water and assigned diets throughout the study. The experiment took place at the National Research-Development Institute for Animal Nutrition and Biology, Balotești, Romania. The local ethics committee approved all experimental procedures in this animal study (*n* = 4 per group). At the end of the 30-day period, in compliance with EU Council Directive 98/58/EC and Romanian Law 206/2004 (Ethics Committee no. 118/02.12.2019), the animals were sacrificed. Liver samples were collected from the middle of the right lobe, as distant as possible from the periportal area, and kidney samples were obtained as triangular segments resulting from cross-sections, with the large base oriented towards the cortex. All samples were stored at −80 °C.

### 2.2. Isolation of the Cytoplasmatic Fraction

The cytoplasmatic fraction isolation method was conducted with modifications of the protocol described by Rasmussen et al. [35]. Six grams of sample tissue were minced and homogenised in ice-cold Tris-sucrose buffer using a Potter homogenizer. The resulting homogenate was centrifuged at 10,000× *g* for 10 min at 4 °C to obtain the crude homogenate without nuclei. This obtained crude homogenate was then centrifuged at 100,000× *g* for 60 min at 4 °C, resulting in the cytoplasmatic fraction in the supernatant and the microsomal fraction in the pellet. The cytoplasmatic supernatant was collected and stored at −80 °C. A purity assay was conducted via immunoblotting (as described previously by Popescu et al. [34]). All procedures were carried out on ice.

### 2.3. Protein Digestion and LC-MS Analysis

The cytoplasmatic fraction obtained was subjected to a proteomic analysis. The Bradford method was used to determine the protein concentration. A quantity of 30 μg of protein was resuspended in 500 μL of 50 mM NH_4_HCO_3_, treated with 25 μL of 100 mM DTT at 37 °C for 45 min, alkylated by adding 26.25 μL of 300 mM IAA, and incubated for another 45 min at 37 °C in the dark. Trypsin digestion was performed with trypsin solution (Trypsin Gold, V528A, Promega, Madison, WI, USA) at a ratio of 1:50 at 37 °C for 16 h. Digestion was terminated by adding 10 μL of 10% trifluoroacetic acid.

The peptides were dried using a speed-vacuum, resuspended in 30 μL of 2% acetonitrile with 0.1% formic acid, and fractionated using a NanoLC 425 system (Eksigent, Dublin, CA, USA) in a trap–elute configuration. This system included a trapping column C18 (5 μm, 300 μM ID, 25 mm length) and an analytical column Eksigent 5C18-CL-120 (300 μM ID, 150 mm length) connected to a DuoSpray ion source (AB Sciex, Framingham, MA, USA). A volume of 5 μL of peptides was loaded onto the trap column at a flow rate of 40 μL /min with 0.1% formic acid and eluted with a gradient from 5 to 80% acetonitrile with 0.1% formic acid over 105 min at a flow rate of 5 μL/min and a column temperature of 55 °C. Ionisation was performed using electrospray ionisation in positive ion mode, with the ion source parameters set up as follows: GS1: 15, GS2: 0, CUR: 25, TEM: 0, and ISVF: 5500 V. The TRIPLE TOF 5600+ operated in Data-Independent Acquisition (DIA) mode, with 64 variable windows (SWATH 64 vw), as previously described [25]. The mass spectrometry proteomics data were deposited in the ProteomeXchange Consortium via the PRIDE [36] partner repository with the dataset identifier PXD050835. All samples in this study were run in triplicate.

### 2.4. Data Analysis

Label-free, library-free protein identification from DIA data was conducted using DIA-NN ver. 1.8.1 [37]; we searched the raw spectra against the fasta file of the complete Sus scrofa proteome (UniProt, UP000008227, January 2024, 46,177), with a precursor m/z range of 400–1250 and trypsin as the digestion enzyme. The data were searched with match between runs (MBR) enabled, robust LC quantification, retention time-dependent normalisation, and a false discovery rate (FDR) < 0.01. Cysteine carbamidomethylation (fixed) and methionine oxidation (variable) modifications were selected. Quantitation was performed using the MaxLFQ algorithm. The unique gene matrix *tsv file from DIA-NN was utilised for performing statistical and differential downstream analysis using the Limma test with locally installed PolySTest version 1.3 [38], available at https://bitbucket.org/veitveit/polystest/src/master/ (accessed on 7 February 2024). For all data, the FDR-adjusted *p* value threshold was set to 0.05 and the log_2_ fold change (log2FC) outside of the (−0.5, 0.5) interval to highlight the most significantly down- and up-regulated proteins. Following statistical analysis, expression profiles, heatmaps, and plots were performed using the R studio platform (version 2023.12.1 + 402) with the org.Ssc.eg.db R package in Bioconductor for *Sus scrofa* [39]. Differential pathway expression analyses (PEAs) were analysed with Pathfind R package, version 1.6.4 [40], using a protein–protein interaction network (PIN) analysis adapted approach, with PIN data for *Sus scrofa* from STRING. For pathway enrichment analysis (PEA), an enrichment chart and term graph, which represented pathways sorted by the lowest *p* value, were generated. Finally, PathfindR output was utilised for integrating and visualising the data for the main biological pathways using Pathview R package version 1.31.1 [41] and the KEGG pathway database. The categorization and visualisation of proteins according to their roles in toxin metabolism, oxidative stress response, specific tissue localization, and those most modified but with still unknown potential were performed in R version 4.1 using an adaptation of the clusterProfiler package version 4.12.0 and functional data from UniProt for the proteome UP000008227.

## 3. Results

To evaluate the impact of AFB1 and OTA exposure and antioxidant supplementation on cytoplasmatic metabolism, we analysed liver and kidney cytoplasmatic fractions from weaned piglets by a proteomic approach. All DIA data were analysed in library-free mode using DIA-NN (version 1.8.1) against the *Sus scrofa* proteome fasta file. We identified a total of 1279 unique proteins and 56,023 precursors within liver cytoplasmatic samples, and 1634 unique proteins and 63,344 precursors within kidney cytoplasmatic samples, at a false discovery rate (FDR) below 0.01. Differential protein expression was assessed by analysing the uniquely identified proteins with the Limma test. The full results are available in the Supplementary Materials (Tables S1 and S2). The relative expression of each protein from each experimental group (A, M, and AM) compared to the control group (C) is shown on KEGG pathways created by the Pathview R module (Supplementary Materials Document S1).

### 3.1. Dietary Supplementation Modulates Toxin Metabolism

**Figure 1:**
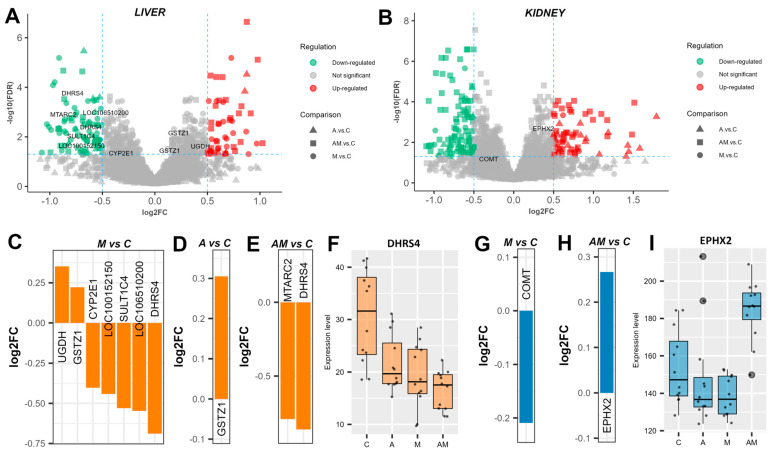
Dietary supplementation modulates toxin metabolism. Volcano plots highlighting proteins involved in toxin metabolism in the liver (**A**) and kidney (**B**) cytoplasmatic fractions, showing log2 fold change (log2FC) for A versus C, M versus C, and AM versus C, with an FDR-adjusted *p* value < 0.05, and log2FC thresholds of −0.5 and 0.5. Bar plots represent the expression level of significantly changed proteins in the liver cytoplasmatic fraction for M versus C (**C**), A versus C (**D**), and AM versus C (**E**). A box plot shows the expression level of dehydrogenase/reductase SDR family member 4 (EC 1.1.1.184, DHRS4) (**F**). Bar plots represent protein expression level of significant changed proteins in the kidney cytoplasmatic fraction for M versus C (**G**), and AM versus C (**H**). A box plot shows the expression levels of cytosolic epoxide hydrolase 2 (EPHX2) (**I**). The C group represents the control group of weaned piglets (**C**) fed with a basal diet. The A group was fed with the basal diet plus a mixture of two antioxidant byproducts (grapeseed and sea buckthorn meal. The M group was fed with the basal diet artificially contaminated with two mycotoxins (AFB1 and OTA). The AM group represents the weaned piglets fed with the basal diet containing the mixture (1:1) of antioxidant byproducts and the two mycotoxins.

In the liver, eight of the identified proteins are known to be involved in toxin metabolism (Figure 1A). Among these, when comparing the group fed with a mycotoxin (M)-contaminated diet, artificially supplemented with AFB1 and OTA, to the control (C) group, UDP-glucose 6-dehydrogenase (EC 1.1.1.22, UGDH) and glutathione S-transferase zeta 1 (GSTZ1) were found to be significantly up-regulated (Figure 1C). On the other hand, the expression levels of cytochrome P450 2E1 (CYP2E1), sulfotransferases (LOC100152150 and SULT1C4), glutathione S-transferase (EC 2.5.1.18, LOC106510200), and dehydrogenase/reductase SDR family member 4 (EC 1.1.1.184, DHRS4) were down-regulated in the M versus C comparison. When comparing the antioxidant-supplemented diet group (A) with the control group (C), only GSTZ1 was slightly up-regulated, but higher than in the M versus C comparison (Figure 1D). In the case of the concomitant administration of antioxidants and mycotoxins (AM) group to the control group (C), only two proteins were differentially expressed, mitochondrial amidoxime reducing component 2 (MTARC2) and DHRS4 (Figure 1E). Interestingly, DHRS4 expression was significantly lower in group M compared with C (log2FC = −0.69), and even lower in group AM (log2FC = −0.86) (Figure 1F). The identified liver proteins involved in toxin metabolism are part of phase I reactions via the drug metabolism pathway (ssa00983, Figure S7) and phase II reactions via glutathione metabolism (ssa00480, Figure S8).

Only two proteins involved in toxin metabolism were identified in the kidneys (Figure 1B). In the M versus C comparison, catechol O-methyltransferase (EC 2.1.1.6, COMT) was down-regulated (Figure 1G). No specific protein expression was significantly changed in the A versus C comparison. In the AM versus C comparison, cytosolic epoxide hydrolase 2 (EPHX2) was up-regulated (Figure 1H), although its expression was down-regulated following the administration of antioxidants or mycotoxins alone in the diet. COMT participates in tyrosine metabolism (ssa00350, Figure S31), whereas EPHX2 is involved in glutathione metabolism (ssa00480, Figure S32).

### 3.2. Antioxidant Proteins Indicate Redox Imbalance Under Exposure to a Mycotoxin-Contaminated Diet

**Figure 2:**
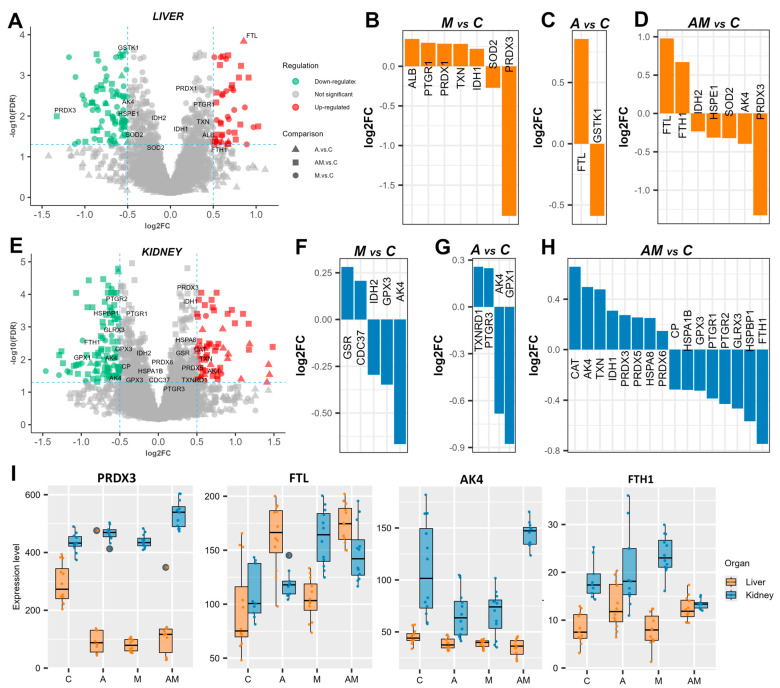
Redox imbalance under exposure to a mycotoxin-contaminated diet. Volcano plots highlighting proteins involved in oxidative stress in the liver (**A**) and kidney (**E**) cytoplasmatic fractions, showing log2 fold change (log2FC) for A versus C, M versus C, and AM versus C, with an FDR-adjusted *p* value < 0.05, and log2FC thresholds of −0.5 and 0.5. Bar plots represent expression levels of significantly changed proteins in the liver cytoplasmatic fraction for M versus C (**B**), A versus C (**C**), and AM versus C (**D**). Bar plots represent protein expression levels of significantly changed proteins in the kidney cytoplasmatic fraction for M versus C (**F**), A versus C (**G**), and AM versus C (**H**). A box plot shows the expression levels of thioredoxin-dependent peroxiredoxin 3 (PRDX3), ferritin light chain (FTL), adenylate kinase 4 (AK4), and ferritin heavy chain (FTH1) (**I**). The C group represents the control group of weaned piglets (**C**) fed with a basal diet. The A group was fed with the basal diet plus a mixture of two antioxidant byproducts (grapeseed and sea buckthorn meal. The M group was fed with the basal diet artificially contaminated with two mycotoxins (AFB1 and OTA). The AM group represents the weaned piglets fed with the basal diet containing the mixture (1:1) of antioxidant byproducts and the two mycotoxins.

Among the proteins identified were several known to be involved in combating oxidative stress and maintaining redox balance. Thirteen unique proteins were detected in the liver (Figure 2A). In the M versus C comparison, a slight up-regulation was observed for albumin (ALB), prostaglandin reductase 1 (EC 1.3.1.48, PTGR1), peroxiredoxin-1 (EC 1.11.1.24, PRDX1), thioredoxin (TXN), and isocitrate dehydrogenase [NADP] (EC 1.1.1.42, IDH1), while superoxide dismutase (EC 1.15.1.1, SOD2) and thioredoxin-dependent peroxiredoxin 3, PRDX3) were down-regulated (Figure 2B). In the A versus C comparison, ferritin light chain (FTL) was up-regulated, and glutathione S-transferase kappa 1 (EC 2.5.1.18, GSTK1) was down-regulated (Figure 2C). In the AM versus C comparison, FTL showed a similar degree of change, along with up-regulated ferritin heavy chain (FTH1) and down-regulated IDH2, chaperonin 10 (HSPE1), SOD2, adenylate kinase 4 (AK4), and PRDX3 (Figure 2D).

The expression of several proteins involved in oxidative stress was also altered in the kidneys (Figure 2E). In the M versus C comparison, glutathione reductase (EC 1.8.1.7, GSR) and Hsp90 co-chaperone Cdc37 (CDC37) were up-regulated, whereas IDH2, glutathione peroxidase (EC 1.11.1.9, GPX3), and AK4 were down-regulated (Figure 2F). In the A versus C comparison, cytoplasmic thioredoxin reductase 1 (EC 1.11.1.2, TXNRD1) and 15-oxoprostaglandin 13-reductase (EC 1.3.1.48, PTGR3) were up-regulated, while AK4 and GPX1 were down-regulated (Figure 2G). The greatest number of differentially expressed proteins was observed in the AM versus C comparison, where catalase (EC 1.11.1.6, CAT), AK4, TXN, IDH1, PRDX3, peroxiredoxin-5 (EC 1.11.1.24, PRDX5), heat shock protein family A (Hsp70) member 8 (HSPA8), and peroxiredoxin-5 (EC 1.11.1.27, PRDX6) are up-regulated, and ceruloplasmin (CP), heat shock 70 kDa protein 1B (HSPA1B), GPX3, prostaglandin reductase 1 (PTGR1), prostaglandin reductase 2 (PTGR2), glutaredoxin 3 (GLRX3), Hsp70-binding protein 1 (HSPBP1), and FTH1 were down-regulated (Figure 2H).

Among these oxidative stress-related proteins, PRDX3 expression was significantly decreased in the liver but increased in the kidneys, while FTL levels were higher in the liver than in the kidneys across the different diets. AK4 was generally down-regulated, except in the kidneys in the AM versus C comparison, where it was increased. Moreover, FTH1 showed the opposite pattern, being strongly down-regulated in the kidneys (Figure 2I).

### 3.3. Dietary Supplementation Influences the Expression of Tissue-Specific Proteins

**Figure 3:**
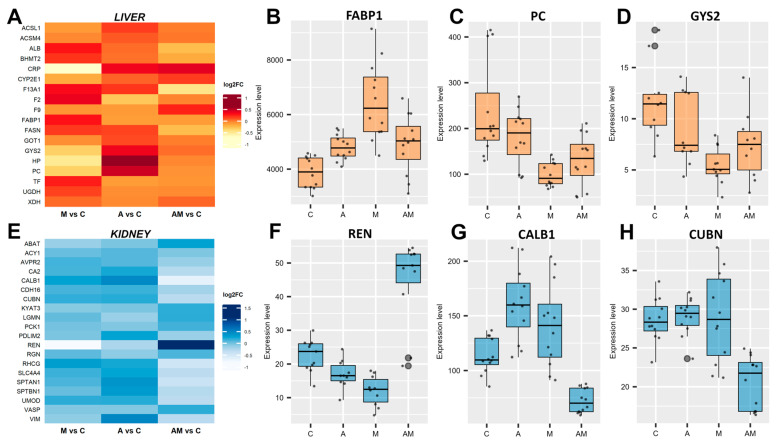
Effects of dietary supplementation on liver and kidney tissue-specific proteins. Heatmap of the differentially expressed proteins, filtered with an FDR-adjusted *p* value < 0.05, in liver (**A**) and kidney (**E**). Box plots show protein expression level (FDR < 0.01) relative to control (**C**) for antioxidant (**A**), mycotoxin (M), and antioxidant + mycotoxin (AM) diets. In liver, fatty acid-binding protein 1 (FABP1) (**B**), pyruvate carboxylase (PC) (**C**), and glycogen synthase 2 (GYS2) (**D**). In the kidney, renin (REN) (**F**), calbindin (CALB1) (**G**), and cubilin (CUBN) (**H**). The C group represents the control group of weaned piglets (**C**) fed with a basal diet. The A group was fed with the basal diet plus a mixture of two antioxidant byproducts (grapeseed and sea buckthorn meal. The M group was fed with the basal diet artificially contaminated with two mycotoxins (AFB1 and OTA). The AM group represents the weaned piglets fed with the basal diet containing the mixture (1:1) of antioxidant byproducts and the two mycotoxins.

In the liver, proteins related to lipid metabolism, glucose storage, and energy regulation were differentially expressed (Figure 3A, Supplementary Materials Document S1, Table S1). Fatty acid-binding protein 1 (FABP1) was consistently up-regulated across all dietary comparisons (Figure 3B), with the highest increase observed in the M versus C group (log2FC = 0.73). In contrast, glycogen synthase 2 (GYS2) and pyruvate carboxylase (PC), both involved in carbohydrate metabolism, were down-regulated under mycotoxin exposure, with the strongest effect observed in M versus C (log2FC = −1.11 for GYS2, and −1.19 for PC) (Figure 3C,D). In addition, fatty acid synthase (FASN) showed reduced expression in the AM group (log2FC = −0.43), suggesting impaired lipogenesis under combined exposure. Also, albumin (ALB) and transferrin (TF) were increased in the M group, while acute-phase proteins (APPs) such as the C-reactive protein (CRP) and coagulation factor XIII (F13A1) were suppressed, suggesting perturbations in hepatic transport and inflammatory response pathways. Together, these hepatic changes indicate alterations in energy metabolism under mycotoxin exposure, which were only partially alleviated by antioxidant supplementation (AM group).

In the kidneys, calbindin (CALB1) expression was increased in both A and M groups but markedly decreased in AM (log2FC = −0.65). Cubilin (CUBN), a receptor essential for protein reabsorption processes, was also down-regulated in the AM group (log2FC = −0.49) (Figure 3H). Interestingly, renin (REN) expression showed variation, being suppressed in the M group (log2FC = −0.98) but strongly induced in the AM group (log2FC = 0.95), suggesting an interactive effect of antioxidants and mycotoxins on the renin–angiotensin system. Beyond these, aminoacylase-1 (ACY1), carbonic anhydrase 2 (CA2), vasodilator-stimulated phosphoprotein (VASP), and regucalcin (RGN) revealed differential protein regulation in kidney amino acid metabolism, acid–base balance, calcium signalling, and vascular regulation (Figure 3E, Supplementary Materials Document S1, Table S2).

### 3.4. Dietary Supplementation and Mycotoxin Exposure Induce Strong Modifications in Liver and Kidney Proteomes with Uncertain Biological Implications

Proteomic analysis of liver and kidney cytoplasmatic fractions revealed changes in the protein expression in response to the mycotoxin-contaminated diet (M group), to antioxidant supplementation (A group), and their concomitant administration (AM group). While many proteins were differentially expressed across comparisons, we focus here on those proteins that show strong modulation but with unclear or unexpected biological significance, especially in pigs as an organism (Tables S1 and S2).

**Figure 4:**
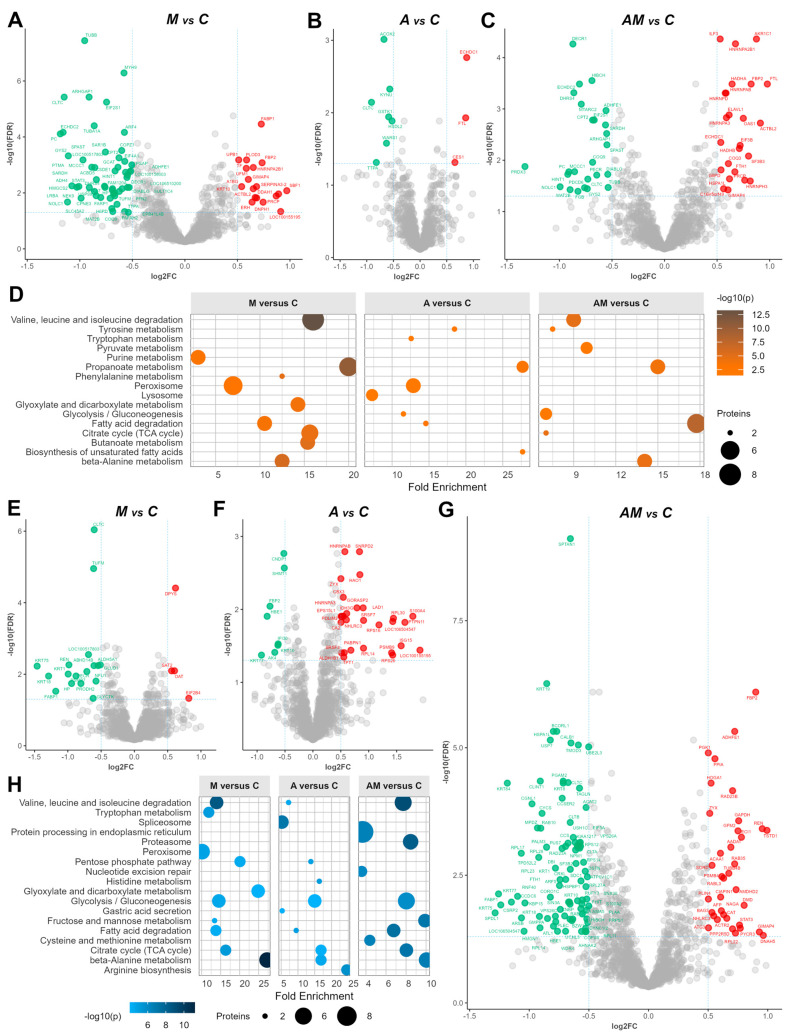
Volcano plots for the liver cytoplasmatic fraction, showing log2FC from M versus C (**A**), A versus C (**B**), and AM versus C (**C**), and for the kidney cytoplasmatic fraction, showing log2FC from M versus C (**E**), A versus C (**F**), and AM versus C (**G**), at an FDR-adjusted *p* value < 0.05, and log2 threshold (−0.5, 0.5). Green indicates significantly down-regulated proteins, red indicates significantly up-regulated proteins, and gray indicates proteins having expression changed below the FDR and log2FC criteria Enrichment chart for the top 10 KEGG pathways in liver (**D**) and kidney (**H**) cytoplasmatic fractions, sorted by the lowest *p* value. The C group represents the control group of weaned piglets (**C**) fed with a basal diet. The A group was fed with the basal diet plus a mixture of two antioxidant byproducts (grapeseed and sea buckthorn meal). The M group was fed with the basal diet artificially contaminated with two mycotoxins (AFB1 and OTA). The AM group represents the weaned piglets fed with the basal diet containing the mixture (1:1) of antioxidant byproducts and the two mycotoxins.

In the liver, a comparison between the M versus C (Figure 4A) showed 76 significant proteins; from these, the largest decreases were observed for acyl-CoA thioesterase 6 (ACOT6, log2FC = −2.60), annexin A6 (ANXA6, log2FC = −2.25), purine nucleoside phosphorylase (PNP, log2FC = −1.77), annexin A4 (ANXA4, log2FC = −1.74), and enoyl-CoA hydratase domain containing 2 (ECHDC2, log2FC = −1.16), while prolylcarboxypeptidase (PRCP, log2FC = 0.87), alpha-1-antichymotrypsin 2 (SERPINA3-2, log2FC = 0.89), guanylate-binding protein 7 (LOC100155195, log2FC = 0.91), and SET binding factor 1 (SBF1, log2FC = 0.97) were up-regulated.

For the comparison between A group and control (C) (Figure 4B), 10 proteins were highlighted, with a slight decrease in the clathrin heavy chain (CLTC, log2FC = −0.91), alpha tocopherol transfer protein (TTPA, log2FC = −0.82), and acyl-CoA oxidase 2 (ACOX2, log2FC = −0.68), whereas ethylmalonyl-CoA decarboxylase 1 (ECHDC1, log2FC = 0.87) and carboxylesterase 1 (CES1, log2FC = 0.65) were up-regulated. Other proteins (Figure S1), including kynureninase (KYNU), tryptophanyl-tRNA synthetase 1 (WARS1), and hydroxysteroid dehydrogenase like 2 (HSDL2), also indicated varying degrees of differential expression, suggesting potential regulatory roles in response to the antioxidant diet.

Concomitant administration (AM versus C) produced the strongest modulation in liver, with 51 proteins being strongly modified (Figure 4C), in which nucleolar and coiled-body phosphoprotein 1 (NOLC1, log2FC = −0.99), histidine triad nucleotide binding protein 1 (HINT1, log2FC = −0.92), and methionine adenosyltransferase 2B (MAT2B, log2FC = −0.89) were down-regulated, whereas SFB1 (log2FC = 1.09) and guanylate-binding protein 7 (LOC100155195, log2FC = 2.49) were up-regulated, suggesting potential compensatory responses or adaptations to the concomitant administration of antioxidants and mycotoxins.

Moreover, enrichment analysis (Figure 4D) revealed that in the M versus C comparison, amino acid degradation and energy metabolism pathways (including valine, leucine and isoleucine degradation, propanoate, beta-alanine, butanoate, phenylalanine, TCA cycle, glyoxylate/dicarboxylate, and purine metabolism) were strongly affected. In the A versus C comparison, propanoate metabolism and related pathways (peroxisome, tryptophan, lysosome, and unsaturated fatty acid biosynthesis) showed the main alterations, while in the AM versus C comparison, central carbon and amino acid metabolism was most enriched, including glycolysis and gluconeogenesis, propanoate metabolism, fatty acid degradation, tyrosine metabolism, pyruvate metabolism, beta-alanine metabolism, TCA cycle, and valine, leucine and isoleucine degradation (Table S3, Figure S2).

In the kidney, the analysis of the log2FC data between the M group and the C group (Figure 4E) showed 23 significantly changed proteins, with the most substantial decrease in expression for acyl-CoA thioesterase 6 (ACOT6, log2FC = −2.20), followed by haptoglobin (HP, log2FC = −0.94) and proteasome 20S subunit beta 7 (PSMB7, log2FC = −0.88) with a slightly lower expression. On the other hand, eukaryotic translation initiation factor 2B subunit delta (EIF2B4, log2FC = 0.82), dihydropyrimidinase (DYPS, log2FC = 0.62), and ornithine aminotransferase (OAT, log2FC = 0.60) were up-regulated.

When kidney cytoplasmatic samples from the A group were compared to those from the control group (C), the log2FC of 41 proteins changed significantly (Figure 4F). Particularly, keratin 77 (KRT77, log2FC = −0.92), haemoglobin subunit epsilon 1 (HBE1, log2FC = −0.82), and fructose-bisphosphatase 2 (FBP2, log2FC = 0.77). Additionally, carbamoyl-phosphate synthase 1 (CPS1) showed the most decreased expression (log2FC = −2.57). On the other hand, 2′-5′-oligoadenylate synthetase 1 (OAS1) and ribosomal protein 2 (RPS2) demonstrated increased expression with log2FC values of 2.13 and 2.12, respectively. Additionally, several other proteins, including lysozyme (LYZ), S100 calcium-binding protein A4 (S100A4), and ISG15 ubiquitin-like modifier (ISG15), displayed varying degrees of up-regulated expression, suggesting potential roles in the kidney’s response to the antioxidant diet.

As in the case of liver samples, we observed the most abundant significant differences between the AM group and the control group (C), for a larger number of proteins, specifically 128 proteins (Figure 4F). Among these, spindle apparatus coiled-coil protein 1 (SPDL1, log2FC = −1.29), ribosomal protein L17 (RPL17, log2FC = −1.07), cingulin like 1 (CGNL1, log2FC = −0.98), and clathrin interactor 1 (CLINT1, log2FC = −0.91) showed the most down-regulation effect (Figure S3). On the other hand, lamin A/C (LMNA) showed the highest up-regulation (log2FC = 2.33). There was also up-regulation in 2′-5′-oligoadenylate synthetase 1 (OAS1, log2FC = 1.51), ISG15 ubiquitin like modifier (ISG15, log2FC = 1.48), thymosin beta 10 (TMSB10, log2FC = 1.10), and thiosulfate sulfurtransferase (TSTD1, log2FC = 0.99). All log-ratios of identified proteins in the kidney cytoplasmatic samples are provided in Table S2.

Enrichment analysis (Figure 4H) revealed that in the M versus C comparison, beta-alanine metabolism showed the strongest enrichment alongside perturbations in valine, leucine, and isoleucine degradation, the TCA cycle, pentose phosphate pathway, and fatty acid degradation. In the A versus C comparison, amino acid-related pathways such as beta-alanine, arginine, and histidine metabolism were most enriched, while in the AM versus C comparison, broader metabolic pathways were highlighted, including valine, leucine, and isoleucine degradation, the TCA cycle, glycolysis, fatty acid degradation, fructose and mannose metabolism, cysteine and methionine metabolism, proteasome, and protein processing in the endoplasmic reticulum (Table S4, Figure S4).

### 3.5. Interaction Effects Reveal Distinct Proteomic Signatures of Antioxidants and Mycotoxins

**Figure 5:**
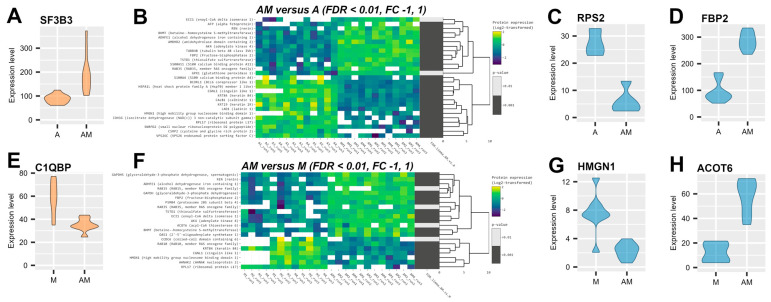
Impact of mycotoxins and antioxidants in the piglet’s diet. Violin plots represent protein expression level of splicing factor 3b subunit 3 (SF3B3) from liver cytoplasmatic samples from A and AM groups (**A**) and complement the C1q binding protein (C1QBP) from M and AM groups (**E**) and from kidney cytoplasmatic samples from ribosomal protein S2 (RPS2) (**C**) and fructose-bisphosphatase 2 (FBP2) (**D**) from A and AM groups, and for high mobility group nucleosome binding domain 1 (HMGN1) (**G**) and acyl-CoA thioesterase 6 (ACOT6) (**H**) from M and AM groups. Clustered heatmap of the differentially expressed proteins from AM versus A (**B**) and from AM versus M (**F**), filtered with the log2FC threshold set to exclude interval (−1, 1) and FDR-adjusted *p* value < 0.01. Yellow colour represents up-regulation, while blue represents down-regulation in the A group compared to the AM group. From left to right, expression values (log2 transformed) for replicates (4 biological × 3 technical) are shown for the A/M group and for the AM group, followed by significance values of the comparison to the A/M group. The M group was fed with the basal diet artificially contaminated with two mycotoxins (AFB1 and OTA). The A group was fed with the basal diet plus a mixture of two antioxidant byproducts (grapeseed and sea buckthorn meal). The AM group represents the weaned piglets fed with the basal diet containing the mixture (1:1) of antioxidant byproducts and the two mycotoxins.

To evaluate the interaction effects of mycotoxins and antioxidants, we compared the AM group to the A group (AM versus A) to identify proteins significantly influenced (*p* value < 0.01) by mycotoxin exposure in the presence of antioxidants. In the liver cytoplasmatic fraction (Table S5), only one protein, splicing factor 3b subunit 3 (SF3B3), showed significant up-regulation (log2FC = 1.03) (Figure 5A), while a less stringent threshold (−0.5, 0.5) highlighted 11 additional proteins with moderate changes (Figure 5B,C). In contrast, kidney cytoplasmatic fractions showed pronounced alterations in the AM versus A comparison (Table S6), with 41 proteins showing significant differential expression (Figure 5D). Up-regulated proteins include enzymes involved in gluconeogenesis (FBP2), amino acid and one-carbon metabolism (BHMT, KYNU, CPS1), energy homeostasis (AK4), vesicle trafficking (RAB15, RAB35), and cytoskeletal integrity (TUBB4B, ACSF3, REN, AFP). Down-regulated proteins were linked to ribosomal function (RPS4X, RPL15, RPS2, RPL30, RPL14, RPL31, RPL17), nucleosome binding (HMGN1), calcium homeostasis (CALB1, S100A4, S100A11), cytoskeletal organisation (CGNL1, LAD1, KRT19, KRT84), endosomal trafficking (VPS26C), protein folding (HSPA1L), and RNA processing (SNRPD2, BCORL1, CCDC6, SPDL1), highlighting a coordinated response to mycotoxin exposure in the presence of antioxidants.

The effect of antioxidant supplementation in the mycotoxin-contaminated diet (AM versus M) was primarily observed in kidney cytoplasmatic fractions (Table S7), with 24 proteins exhibiting significant differential expression (log2FC outside −1, 1) (Figure 5F), whereas changes in the liver cytoplasmatic fraction were modest (Table S8), with only 12 proteins, including SF3B3 and complement C1q binding protein (C1QBP), detected at a lower threshold (log2FC ± 0.5) (Figure 5E). In the kidney, differentially expressed proteins clustered into 16 up-regulated and 8 down-regulated proteins. Up-regulated proteins included metabolic and energy-related enzymes (AK4, BHMT, FBP2, ECI1, ADHFE1) and proteins involved in lipid metabolism and vesicle trafficking (ACOT6, RAB15, RAB35, GAPDH, GAPDHS, PSMB4, REN, TSTD1, OAS1, TOLLIP, ISG15), several of which were distinct from the mycotoxin effect. Down-regulated proteins (HMGN1, RPL17, KRT84, CGNL1, CCDC6, SPDL1, AHNAK2, RAB10) were associated with ribosomal function, nucleosome binding, cytoskeletal organisation, and calcium homeostasis, reflecting both mycotoxin-induced and antioxidant-specific responses.

These results indicate that mycotoxins exert a stronger effect on kidney proteomes compared to the liver under antioxidant supplementation, while antioxidants selectively modulate protein expression, partially counteracting or amplifying the mycotoxin-induced changes.

## 4. Discussion

The balance between detoxification and toxic action depends on the dose and the possibility of saturation of metabolic pathways, individual genetic variation, or species. Tissue differences in the isoenzyme pattern are influenced by age, diet, health status, and sex. In this study, we aimed to shed light on the potential benefits of antioxidant supplementation in mitigating mycotoxin toxicity on liver and kidney function. Rather than focusing on individual proteins or predefined pathways, the availability of DIA methods now allows us to capture a more complete picture of cytoplasmic proteome changes. Our library-free DIA proteomics approach using DIA-NN [31] allowed high-throughput quantification of liver and kidney proteins without relying on pre-existing spectral libraries. Compared to traditional methods such as 2D gels or targeted LC–MS/MS, this approach provided broader proteome coverage, higher reproducibility, and the ability to detect subtle or unexpected changes, including those induced by combined antioxidant and mycotoxin exposure. This methodological advantage represents a novel contribution to pig proteomics and provides an opportunity to visualise, in a physiologically relevant animal model, the impact of antioxidant interventions that are often overlooked in the modern era.

When interpreting the observed proteomic changes, it is important to note that the experimental mycotoxin concentrations are above current EU regulation limits and EFSA reference values. For AFB1, maximum contents in pig feed are generally 10–20 µg/kg [42,43], while guidance values for OTA range from 50 to 250 µg/kg depending on the feed material [44,45]. In this study, the mycotoxin concentrations used, 62 µg/kg of AFB1 and 479 µg/kg of OTA, represent an acute-exposure scenario compared with EU guidance. Therefore, the proteomic alterations reported here should be interpreted in this high-exposure context. This framing supports the study’s aim to investigate whether antioxidant supplementation can mitigate the disruptions induced by high-dose mycotoxin exposure in liver and kidney proteomes. In the liver cytoplasmic fraction, dietary antioxidants and exposure to mycotoxin-contaminated feed showed distinct proteomic alterations. Under mycotoxin challenge (M vs. C), some of the most strongly down-regulated proteins included acyl-CoA thioesterase 6 (ACOT6) and annexin A6 (ANXA6) (Table S1), both of which are associated with fatty acid metabolism and membrane dynamics [46,47,48]. Additional reductions were observed in mitochondrial antioxidant defence (peroxiredoxin 3 as PRDX3) and acute-phase response (C-reactive protein as CRP), suggesting impaired redox homeostasis and inflammatory capacity [49,50]. In contrast, the antioxidant-supplemented diet (A vs. C) mainly reduced acyl-CoA oxidase 2 (ACOX2), a key peroxisomal enzyme in fatty acid β-oxidation, while increasing ferritin light chain (FTL) and ethylmalonyl-CoA decarboxylase 1 (ECHDC1), highlighting adjustments in iron storage and branched-chain fatty acid metabolism [51,52,53].

These findings align with mycotoxin metabolism through the xenobiotic biotransformation pathway, involving their conversion and intracellular translocation, allowing the transformation of toxic substances into compounds suitable for excretion [54]. Therefore, the detoxification process occurs in three phases: modification, or phase I, characterised by the addition of functional groups; conjugation or phase II, involving the conjugation of functional groups; and excretion or phase III. Common phase I reactions include oxidation, reduction, hydrolysis, hydration, and dehalogenation. During this phase, mycotoxins are initially transformed into a more water-soluble chemical form that can be metabolised by the enzymes involved in phase II [55,56]. As we previously observed by proteomic analysis of the microsomal fraction from the liver [25], cytochromes P450 isoforms have a central role in catalysing phase I reactions.

The co-administration of antioxidants and mycotoxins (AM versus C) induced significant changes in the expression of several proteins in the liver cytoplasmatic fraction. Among the most strongly down-regulated proteins were histidine triad nucleotide binding protein 1 (HINT1), which participates in DNA repair [57], and pyruvate carboxylase (PC), which plays a central role in hepatic gluconeogenesis and lipogenesis by catalysing the carboxylation of pyruvate to oxaloacetate, a key intermediate in glucose and lipid metabolism [58]. Additionally, PC contributes to the restoration of the citric acid cycle intermediates and maintains metabolic flux in the liver [59]. The reduction in PC protein expression (Figure S10) suggests a potential impairment in gluconeogenesis and lipogenesis, which are essential for energy production and lipid metabolism in the liver. Additionally, PRDX3 (log2FC = −1.33) was markedly reduced, indicating compromised mitochondrial antioxidant defences. Conversely, several proteins were strongly up-regulated. Guanylate-binding protein 7 (LOC100155195), a member of the GBP family, showed the most pronounced increase, pointing to activation of immune signalling and stress responses [60]. Ferritin light (FTL) and heavy chains (FTH1) were also elevated, consistent with adjustments in iron storage and oxidative stress regulation [61]. Moreover, up-regulation of RNA-binding proteins such as heterogeneous nuclear ribonucleoproteins (HNRNPD, HNRNPAB, HNRNPA3, HNRNPA2B1) suggests reprogramming of mRNA processing and translational control under combined exposure [62]. Enrichment analysis further supports these findings, showing changes in pathways related to propanoate metabolism (Figure S11), fatty acid degradation, and others (Figures S13–S18), indicating the complex regulatory mechanisms that support liver function in response to dietary factors.

Phase II reactions, such as glucuronidation, sulfation, and conjugation with glutathione (GSH), occur primally in the cytoplasm and are the most important in xenobiotic toxicity. The cofactors utilised in these conjunction reactions are uridine 5′-diphosphoglucuronic acid (UDPGA), 3′-Phosphoadenosine-5′-phosphosulfate (PAPS), and GSH. UDP-glucuronosyltransferases (microsomal) and sulfotransferases (cytoplasmatic) catalyse glucuronidation and sulfation reactions, respectively. These enzymes have tissue and species-specific isoforms. For example, in pigs’ liver, specific markers for UDP-glucuronosyltransferases and sulfotransferases are UGT1A1, UGT1A3, UGT1A6, UGT1A10, UGT2B18-like, UGT2B31, UGT2B31-like, SLUT1A1, and SLUT2A1. The resulting glucuronide and sulphate conjugates increase the water solubility of the parent compounds and facilitate their excretion through urine and bile. However, there are cases where phase II detoxification reactions can lead to increased toxicity, as seen in bilirubin glucuronidation by UGT1A1. For instance, fumonisin B1 competes with bilirubin for UGT1A1, leading to hyperbilirubinemia due to enzyme activity inhibition by substrate competition. According to our results, the most affected protein expression levels associated with glucuronidation and sulfation included acyl-CoA thioesterase 6 (ACOT6), aldo-keto reductase family 1 member C1 (AKR1C1), renin (REN), and fatty acid binding protein 1 (FABP1). Other phase II reactions include acetylation catalysed by N-acetyltransferases, methylation of nitrogen (N), oxygen (O), sulphur (S), and arsenic (As) mediated by methyltransferases, and conjugation with amino acids [11,12,63,64,65].

Moreover, phase II reactions provide cellular protection against oxidative stress through the action of antioxidant enzymes: CAT, SOD, and GST (Figure S9). However, under conditions of increased toxicity, the products of phase II may induce changes in protein expression, reactive oxygen species (ROS) production, and oxidative stress in the affected cells [66,67,68,69]. Also, our differential analysis highlights specific proteins involved in antioxidant defence mechanisms and ROS production (Figure S9), such as catalase (CAT), renin (REN), hydroxysteroid dehydrogenase like 2 (HSDL2), glutathione S-transferase kappa 1 (GSTK1), and fatty acid binding protein 1 (FABP1).

Additionally, comparison between the mycotoxin-contaminated diet group (M group) and the control group (C) (Table S2, Figure 4E) in the kidney cytoplasmatic fraction showed a significant down-regulation of acyl-CoA thioesterase 6 (ACOT6), renin (REN), and other proteins, along with up-regulation of dihydropyrimidinase (DYPS). ACOT6 catalyses the hydrolysis of acyl-CoA thioesters to free fatty acids and coenzyme A, regulating intracellular levels of fatty acids [70]. In the kidney, ACOT6 is involved in lipid biosynthesis, maintaining lipid homeostasis. Renin, generated and secreted by kidney juxtaglomerular cells, is a key enzyme in the renin–angiotensin–aldosterone system (RAAS), and it regulates blood pressure and fluid–electrolyte balance by catalysing the conversion of angiotensinogen to angiotensin I, which produces vasoconstrictor angiotensin II. Aldosterone, released by angiotensin II, promotes sodium and water retention, which influences blood pressure and kidney function [71]. While the role of DYPS in the kidney is not fully understood, it is involved in nucleic acid synthesis (Figure S24) and beta-alanine metabolism (Figure S21). These changes may indicate an adaptive response of the kidney to mitigate the effects of mycotoxin-induced stress or direct toxicity to renal cells, highlighting the intricate interplay between environmental exposures and renal function.

On the other hand, for A versus C comparison in the kidney cytoplasmatic fraction, our results revealed a significant decrease in protein expression associated with amino acid metabolism in response to the antioxidant-enriched diet (Table S2, Figure 4F). Specifically, down-regulation of carnosine dipeptidase 1 (CNDP1), involved in arginine, proline (Figure S19), histidine (Figure S20), and beta-alanine metabolism (Figure S21). Also, serine hydroxy-methyltransferase 1 (SHMT1), an essential enzyme in the carbon pool by folate (Figure S22) and biosynthesis of amino acids, and carbamoyl-phosphate synthase 1 (CPS1), a key enzyme in nitrogen metabolism and the urea cycle (Figure S23), indicate alterations in these metabolic pathways. In contrast, we observed increased expression of 2′-5′-oligoadenylate synthetase 1 (OAS1), a protein involved in the immune response [72], and S100 calcium-binding protein A4 (S100A4), often associated with inflammation and cell migration [73], which might indicate a response to the antioxidant diet.

When the concomitant administration of antioxidants and mycotoxins (AM group) was evaluated (Figure 4G), significant alterations in protein expression were observed. This included down-regulation of spindle apparatus coiled-coil protein 1 (SPDL1) and fatty acid binding protein 1 (FABP1), along with the up-regulation of OAS1 and REN, suggesting complex interactions between antioxidants and mycotoxins. In the kidney cytoplasmatic fraction, SPDL1 contributes to the maintenance of genomic stability during cell division [74], ensuring proper development and function of renal cells. FABP1, primarily expressed in the liver but also found in renal proximal tubular cells, is involved in the PPAR signalling pathway (Figure S25), through uptake, transport, and utilisation of fatty acids, which are essential for energy production and membrane synthesis in renal cells [75]. The down-regulation of SPDL1 and FABP1 expression might indicate disruptions in the cell division processes and lipid metabolism, respectively, while the up-regulation of REN suggests potential involvement in the urea cycle. Enrichment analysis (Figure 4H) further highlighted changes in pathways associated with amino acid metabolism, fatty acid degradation (Figure S26), and glycolysis (Figure S27), indicating a multifaceted impact of dietary factors on kidney function and metabolism.

The body eliminates unwanted substances by excretion, which plays an important role in preventing cellular damage and toxicity. Metabolites resulting from phases I and II of detoxification are mainly excreted via urine and bile, while gases are eliminated by exhalation. Also, xenobiotic metabolites can be excreted through sweat, saliva, tears, and breast milk [12,76]. Although the kidneys constitute around 4% of body mass, they receive almost 25% of cardiac output, representing the major route in eliminating compounds and electrolytes via urinary excretion. Glomerular filtration is influenced by the pressure differences between the afferent and efferent arterioles, capillary pore size, and the degree of xenobiotic binding to plasma proteins. Therefore, glomerular filtration allows the passage of molecules below 60 kDa, which depends on the chemical nature of the compounds undergoing excretion and the number of available nephrons. Once filtered, a compound may be excreted or reabsorbed, mainly in the proximal tubules by passive diffusion, depending on metabolite solubility and ionisation. Additionally, xenobiotics can be excreted by an active mechanism mediated by transporters [77,78,79]. According to our results, proteins such as renin, acyl-CoA thioesterase 6, and fatty acid binding protein 1 (FABP1) from the kidney cytoplasmatic fraction might play a role in the transport of xenobiotics.

The interaction between antioxidants and mycotoxins revealed tissue- and pathway-specific proteomic responses, with the kidney as the primary site of modulation. In the AM versus A comparison, in the liver, only the splicing factor 3b subunit 3 (SF3B3) (Table S5, Figure 5A), a complex responsible for pre-mRNA splicing, showed significant up-regulation, which may affect mRNA splicing patterns and potentially affect the expression of genes involved in stress response. Following a less stringent fold change interval, kynureninase (KYNU), hydroxyacyl-CoA dehydrogenase trifunctional multienzyme complex subunit alpha (HADHA), superoxide dismutase 2 (SOD2), and 3-hydroxyisobutyryl-CoA hydrolase (HIBCH) showed significant metabolic adaptations. KYNU catalyses the conversion of 3-hydroxy-L-kynurenine to 3-hydroxyanthranilate, a key step in the degradation of tryptophan (Figure S14). Additionally, tryptophanyl-tRNA synthetase 1 (WARS1) catalyses the attachment of tryptophan to its corresponding tRNA, ensuring accurate incorporation of tryptophan into nascent polypeptide chains during translation [80]. Thus, WARS1 down-regulation may impact translation as a response to changes in tryptophan availability. HADHA catalyses the third step of β-oxidation, converting 3-hydroxyacyl-CoA to 3-ketoacyl-CoA. HADHA expression in liver cells is restored when mycotoxins and antioxidants are co-administered (Figures S12, S16, and S17).

Renal function was severely impacted by the presence of mycotoxins, with significant protein expression changes (Figure 5B). Up-regulation of FBP2 shows an enhanced capacity for gluconeogenesis, possibly to maintain glucose homeostasis in response to mycotoxins and antioxidant exposure (Figure 5D). Also, up-regulation of REN (Figure 3F) may suggest activation of RAAS and a role in metabolic homeostasis. Down-regulation of HMGN1 (Figure 5B), a chromatin-associated protein that modulates chromatin structure and gene expression by binding to nucleosomes [81], could suggest an impact on DNA replication, repair, and transcription. This observation is supported by the down-regulation of RPL17, a component of the ribosome (Figure S28), which suggests an impairment in the protein synthesis machinery. Furthermore, proteins involved in cytoskeletal organisation, chaperones, calcium homeostasis, and nucleic acid binding were affected (Table S6), demonstrating the complex cellular response to combined dietary exposures in renal function.

The impact of antioxidants in the diet contaminated with mycotoxins (AM versus M) was mainly observed in kidney cytoplasmatic samples (Table S7). Specifically, several proteins showed similar expression changes to those induced by mycotoxin exposure alone, suggesting overlapping effects (Figure 5F). Therefore, ADHFE1 and ECI1, involved in fatty acid degradation (Figure S26), AK4, responsible for balancing purine metabolism (Figure S24), BHMT, an enzyme involved in methylation reactions of phase II [82] and in cysteine and methionine metabolism (Figure S29), RAB35, involved in intracellular vesicle trafficking [83], and TSTD1, with a role in the sulphide oxidation pathway [84], may reflect adaptations to maintain energy homeostasis and alterations in response to oxidative stress. However, FBP2 and REN showed unique expression profiles, indicating a potential specific effect of antioxidants. Furthermore, the up-regulated proteins were linked to lipid metabolism (ACOT6) (Figure 5H), energy metabolism (GAPDH, Figure S27), protein turnover (proteasome 20S subunit beta 4—PSMB4, Figure S30), and RAB15 with a role in membrane traffic from the early endosome to the recycling endosome [85], indicating diverse cellular responses to antioxidant exposure. On the other hand, down-regulated proteins in the second cluster (Figure 5F), including AHNAK nucleoprotein 2 (AHNAK2), coiled-coil domain containing 6 (CCDC6), and RAB10, member RAS oncogene family (RAB10), highlighted additional pathways affected by antioxidant intervention, respectively, immune response [86], DNA damage checkpoints [87], and vesicular trafficking [88]. This down-regulation highlights the antioxidant capacity to counteract mycotoxin effects and prevent tumoral cell progression. Liver proteomes were relatively modestly affected (Table S8), with only SF3B3 and C1QBP (Figure 5E) showing minor changes, indicating tissue-specific susceptibility.

The identified effects of antioxidants, as revealed through the alterations in protein expression profiles within the kidney cytoplasmatic fraction, highlight a direct relationship between dietary components and metabolic balance. Although some proteins showed unique expression patterns in the presence of the antioxidants alone, potentially indicating specific effects, the overlapping changes with exposure to mycotoxins suggest a synergic interaction within renal cellular processes.

The results of this study highlight the need to understand the effects determined by supplementing the diet with a mixture of antioxidants rather than a single antioxidant. Cellular metabolism acts differently when exposed to mixtures compared to individual treatments, as the synergistic and/or antagonistic effects are not well understood. To propose solutions that could mitigate or counteract the effects of the mycotoxin contamination in the animal feed, it is important to understand the big picture regarding mechanisms and interactions.

However, our findings are subject to limitations, mainly due to the concentrations of mycotoxins and byproducts containing antioxidants and included in the diet. Additionally, the present study only evaluated the effects of AFB1 and OTA mycotoxins. The biotransformation of xenobiotics depends on the compound structure, physicochemical properties, and the available enzymes in the exposed tissue. Therefore, future research is needed to confirm dose-dependent effects and explore the influence of other types of mycotoxins and antioxidants.

## 5. Conclusions

This study started from the hypothesis that antioxidants can mitigate the adverse effects of mycotoxin contamination on weaned piglets. It was observed that the effects of mycotoxins are partially mitigated by the antioxidant-enriched diet. Our findings show an expression balance of the proteins involved in the amino acid metabolism and antioxidant defence system, accompanied by an energetic compensation through glycolysis and gluconeogenesis. Additionally, we show that in kidneys, some of the effects are synergistically amplified, such as proteins involved in fatty acid degradation, peroxisome, PPAR signalling, translation, the TCA cycle, and the excretion pathways. Inclusion of antioxidants in the animal diet can have beneficial effects. Nevertheless, caution is advised; synergistic effects can occur with potentially more serious consequences than the effect of mycotoxins alone.

## Data Availability

The data presented in this study are available on request from the corresponding author. The mass spectrometry proteomics data have been deposited to the ProteomeXchange Consortium via the PRIDE [30] partner repository with the dataset identifier PXD050835.

## Supplementary Materials

The following supporting information can be downloaded at: https://www.mdpi.com/article/10.3390/antiox14101216/s1, Table S1: Pig liver cytoplasmatic fraction—quantitative proteomic data after Limma test; Table S2: Pig kidney cytoplasmatic fraction—quantitative proteomic data after Limma test; Table S3: Top 10 KEGG Pathways data in liver cytoplasmatic fraction, sorted by lowest p value; Table S4: Top 10 KEGG Pathways data in kidney cytoplasmatic fraction, sorted by lowest p value; Table S5: Pig liver cytoplasmatic fraction—quantitative proteomic data after Limma test for AM vs. A; Table S6: Pig kidney cytoplasmatic fraction—quantitative proteomic data after Limma test for AM vs. A; Table S7: Pig kidney cytoplasmatic fraction—quantitative proteomic data after Limma test for AM vs. M; Table S8: Pig liver cytoplasmatic fraction—quantitative proteomic data after Limma test for AM vs. M; Document S1: Figures S1–S32.
