# Supplementary material for: Proteomic Changes in the Cytoplasmatic Fraction of Weaned Piglets’ Liver and Kidney Under Antioxidant and Mycotoxin Diets
Source: Antioxidants (Basel). 2025 Oct 9;14(10):1216. doi: 10.3390/antiox14101216 (PMC12561714; doi:10.3390/antiox14101216)
Supplement: Supplementary file 1 [file antioxidants-14-01216-s001.zip › Document_S1_v2.pdf]

Figure S1

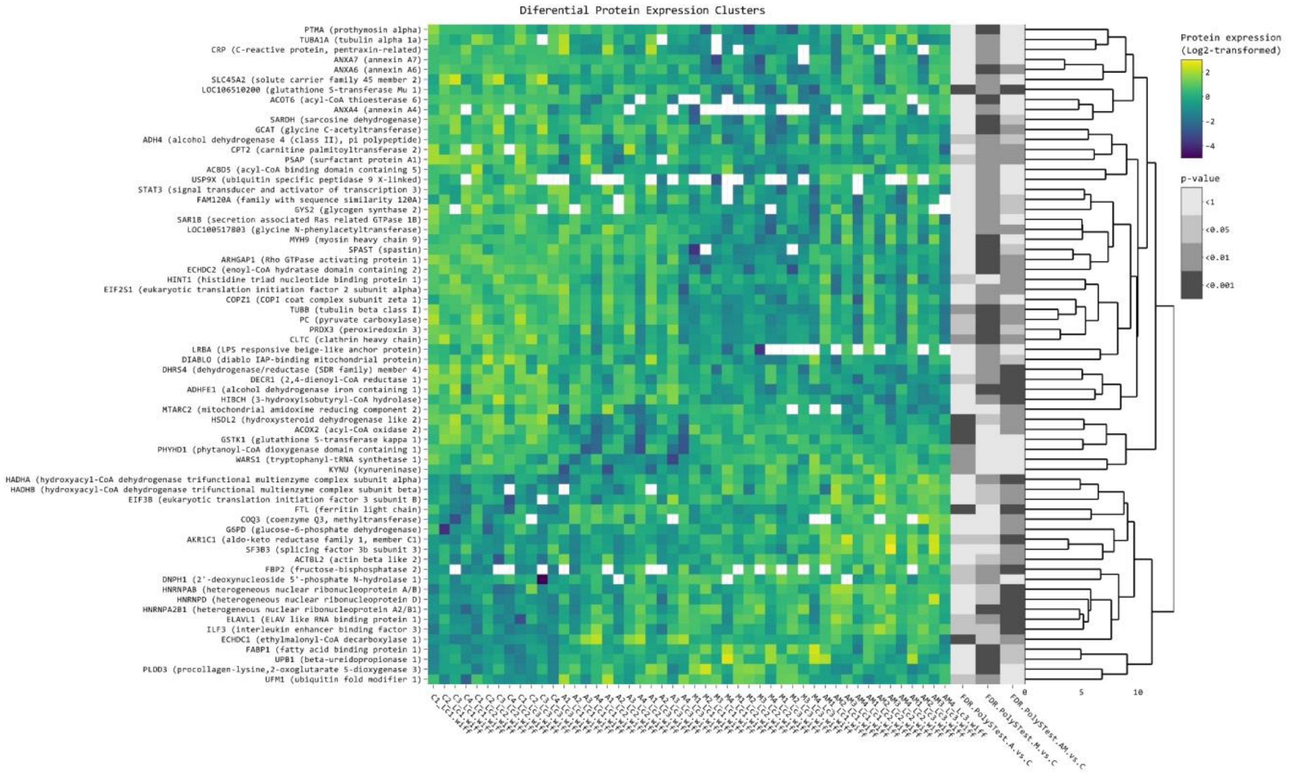

Figure S1. Clustered heatmap of the differentially expressed proteins in liver cytoplasmatic fraction, filtered with log2FC threshold set to exclude interval (-0.5, 0.5) and FDR adjusted p value < 0.01. Yellow colour represents upregulation, while blue represents downregulation in A, M, and AM group compared to Control (C) group. From left to right, expression values (log2 transformed) for replicates (4 biological × 3 technical) are shown for the A, M, and AM groups, followed by significance values of the A versus C, M versus C, and AM versus C comparisons. The control group (C) was fed with a standard diet for starter piglets. The A group were fed with the basal diet plus a mixture of two antioxidant byproducts (grapeseed and sea buckthorn meal. The M group were fed with the basal diet artificially contaminated with two mycotoxins (AFB1 and OTA). The AM group represent the weaned piglets fed with the basal diet containing the mixture (1:1) of antioxidant byproducts and the two mycotoxins.

Figure S2

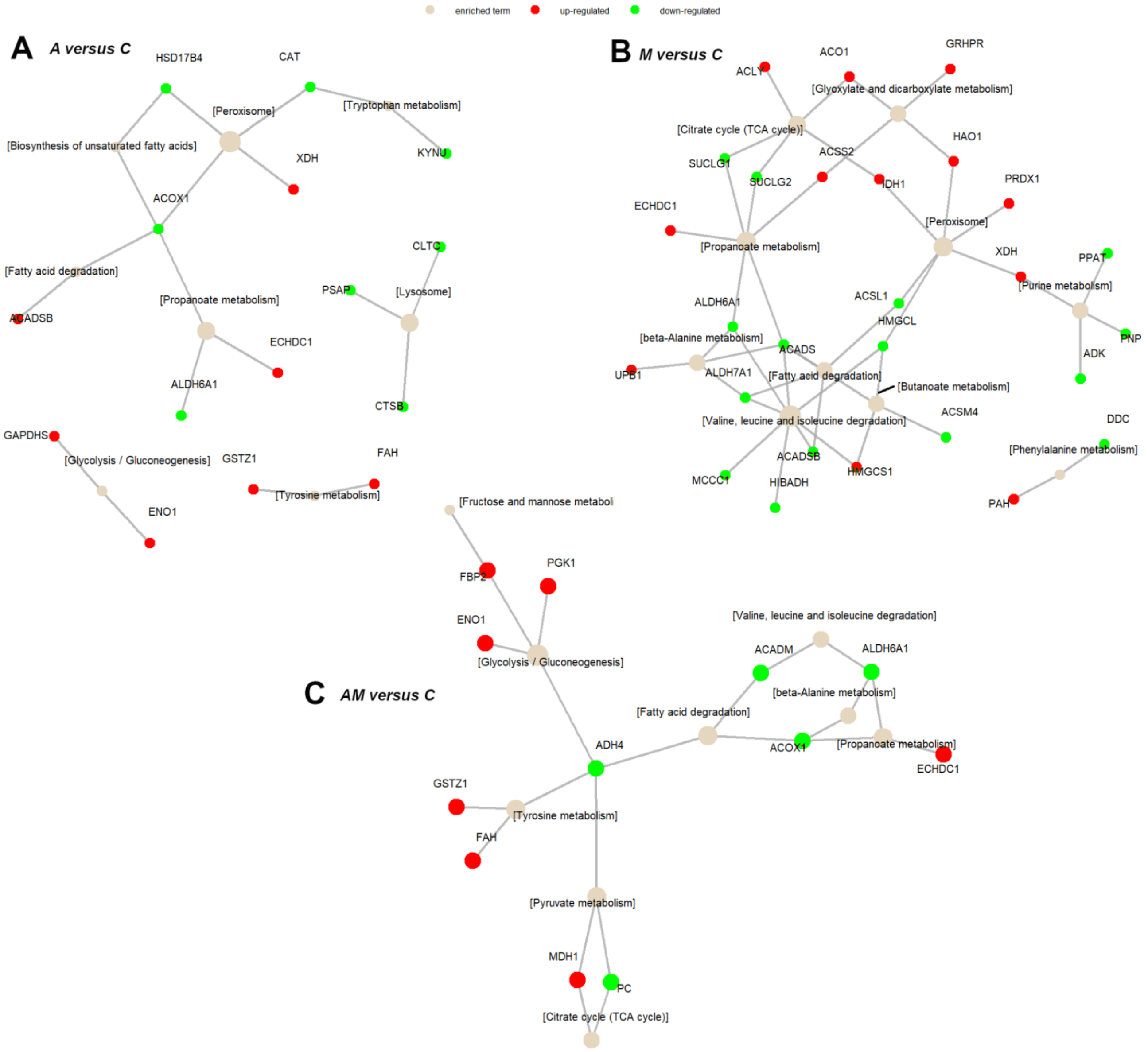

Figure S2. Term-gene graph for top 10 terms in liver cytoplasmatic fraction.

Figure S3

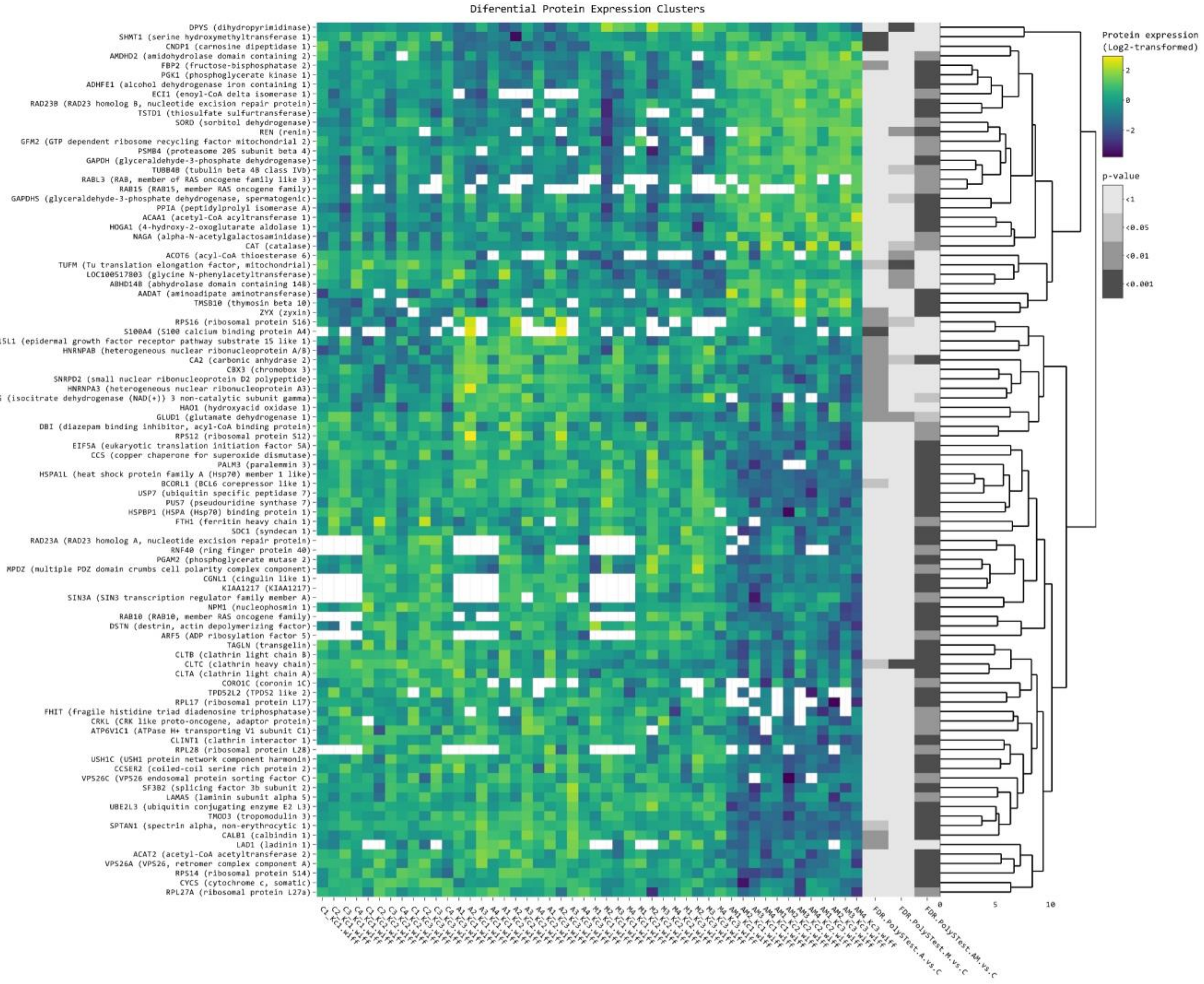

Figure S3. Clustered heatmap of the differentially expressed proteins in kideny cytoplasmatic fraction, filtered with log2FC threshold set to exclude interval (-0.5, 0.5) and FDR adjusted *p* value < 0.01. Yellow colour represents upregulation, while blue represents downregulation in A, M, and AM group compared to Control (C) group. From left to right, expression values (log2 transformed) for replicates (4 biological × 3 technical) are shown for the A, M, and AM groups, followed by significance values of the A versus C, M versus C, and AM versus C comparisons. The control group (C) was fed with a standard diet for starter piglets. The A group were fed with the basal diet plus a mixture of two antioxidant byproducts (grapeseed and sea buckthorn meal. The M group were fed with the basal diet artificially contaminated with two mycotoxins (AFB1 and OTA). The AM group represent the weaned piglets fed with the basal diet containing the mixture (1:1) of antioxidant byproducts and the two mycotoxins

Figure S4

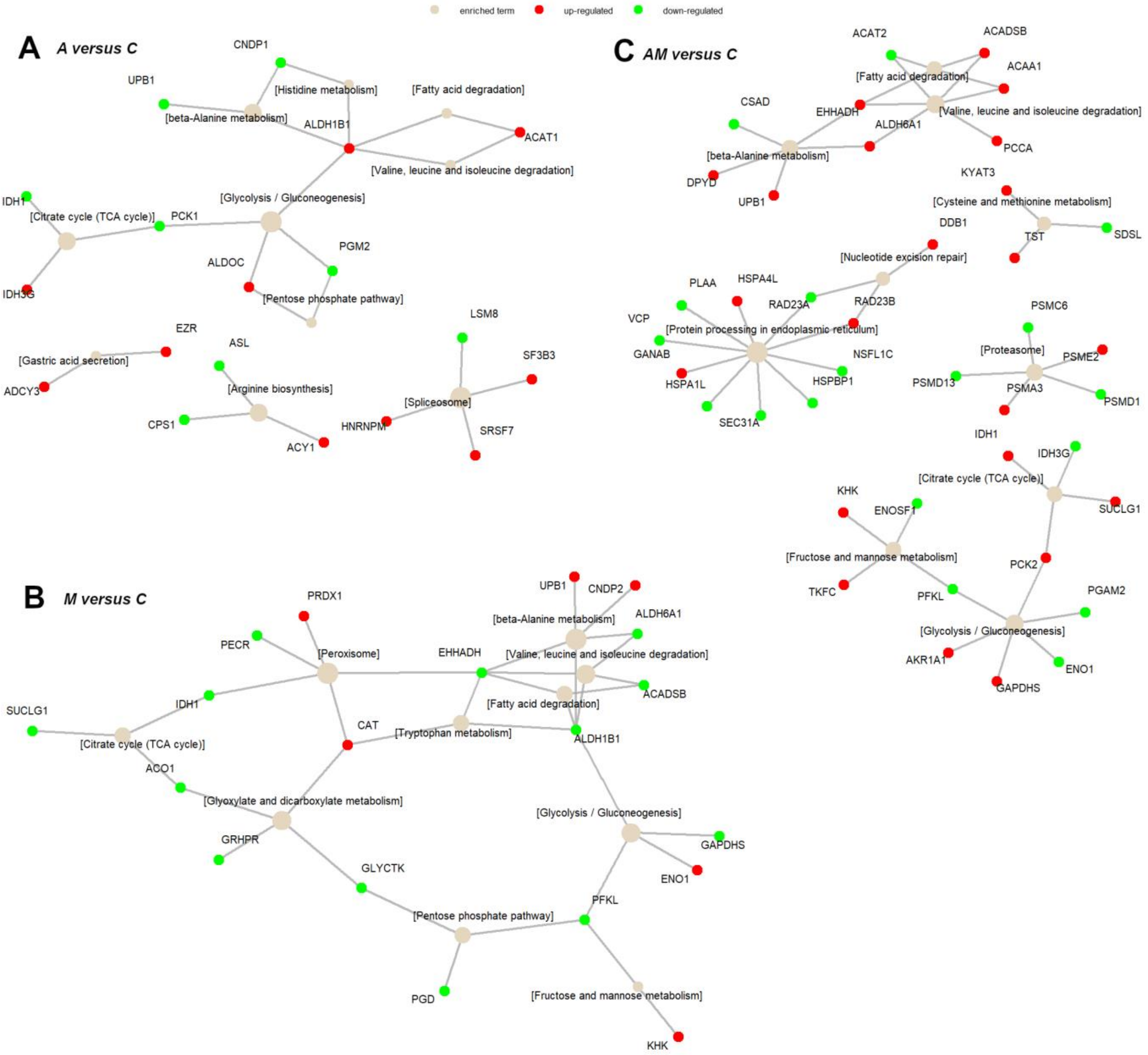

Figure S4. Term-gene graph for top 10 terms in kidney cytoplasmatic fraction.

Figure S5

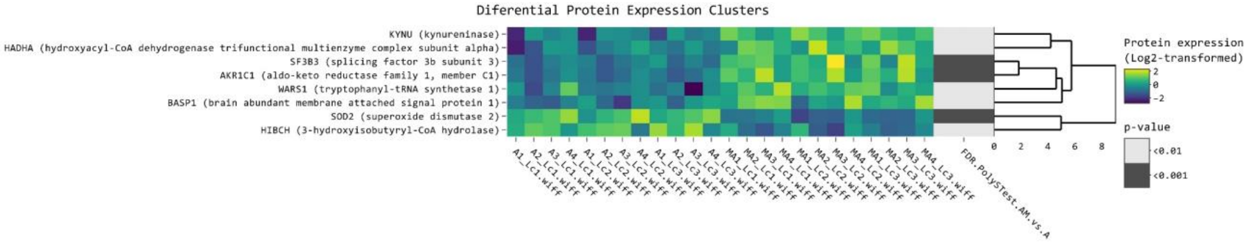

Figure S5. Mycotoxins impact in liver cytoplasmatic fraction. Clustered heatmap of the differentially expressed proteins (AM versus A), filtered with log2FC threshold set to exclude interval (-0.5, 0.5) and FDR adjusted *p* value < 0.01. Yellow colour represents upregulation, while blue represents downregulation in A group compared to AM group. From left to right, expression values (log2 transformed) for replicates (4 biological × 3 technical) are shown for the A group and for the AM group, followed by significance values of the comparison to A group. The A group were fed with the basal diet plus a mixture of two antioxidant byproducts (grapeseed and sea buckthorn meal. The AM group represent the weaned piglets fed with the basal diet containing the mixture (1:1) of antioxidant byproducts and the two mycotoxins.

Figure S6

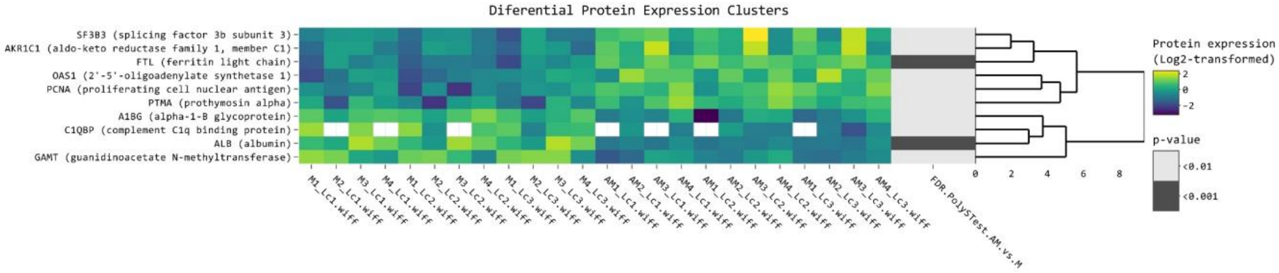

Figure S6. Antioxidants impact in liver cytoplasmatic fraction. Clustered heatmap of the differentially expressed proteins (AM versus M), filtered with log2FC threshold set to exclude interval (-0.5, 0.5) and FDR adjusted *p* value < 0.01. Yellow colour represents upregulation, while blue represents downregulation in A group compared to AM group. From left to right, expression values (log2 transformed) for replicates (4 biological × 3 technical) are shown for the M group and for the AM group, followed by significance values of the comparison to M group. The M group were fed with the basal diet artificially contaminated with two mycotoxins (AFB1 and OTA). The AM group represent the weaned piglets fed with the basal diet containing the mixture (1:1) of antioxidant byproducts and the two mycotoxins.

Figure S7

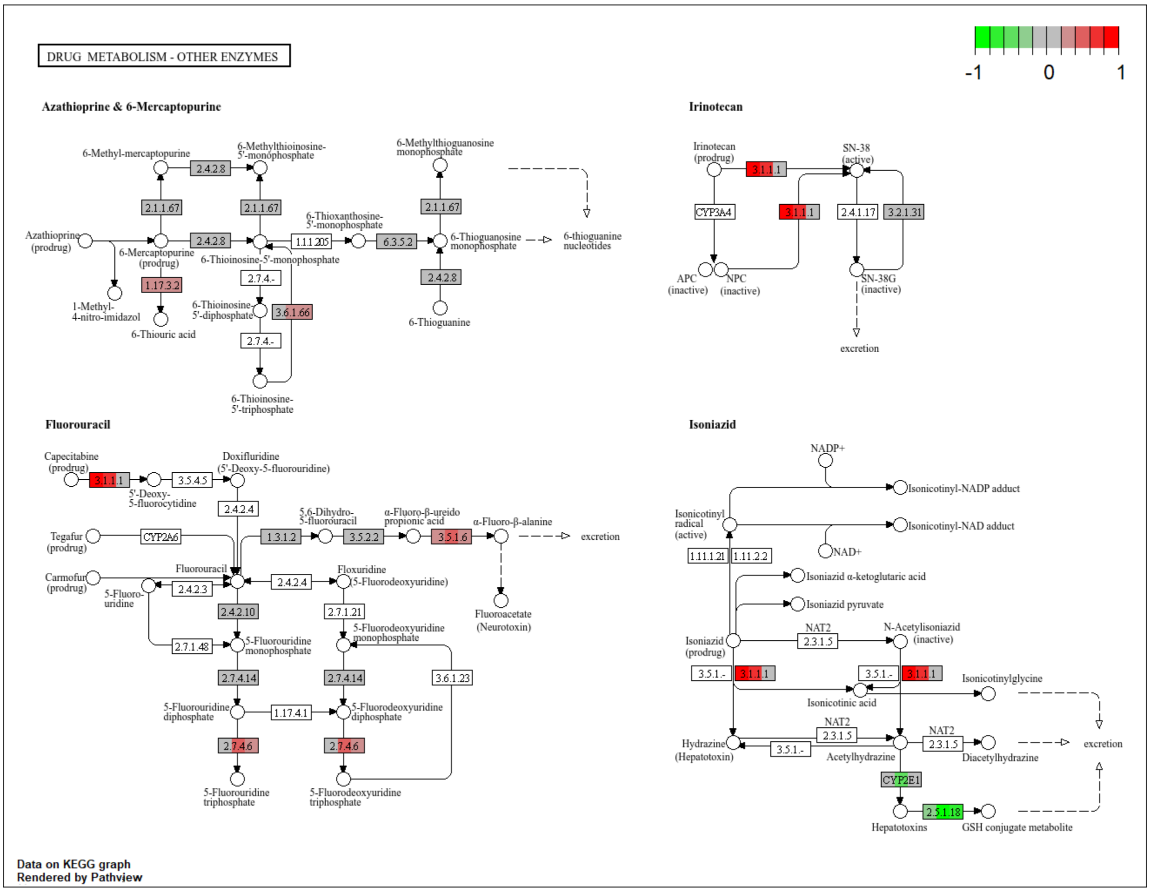

Figure S7. The KEGG drug metabolism pathway for the cytoplasmatic fraction of the liver. The colour of the boxes represents the $\log_2$ fold change in the protein abundances, represented simultaneously for all three comparisons, on the left for A vs. C, in the middle for M vs. C and on the right for AM vs. C in the corresponding box for each protein.

Figure S8

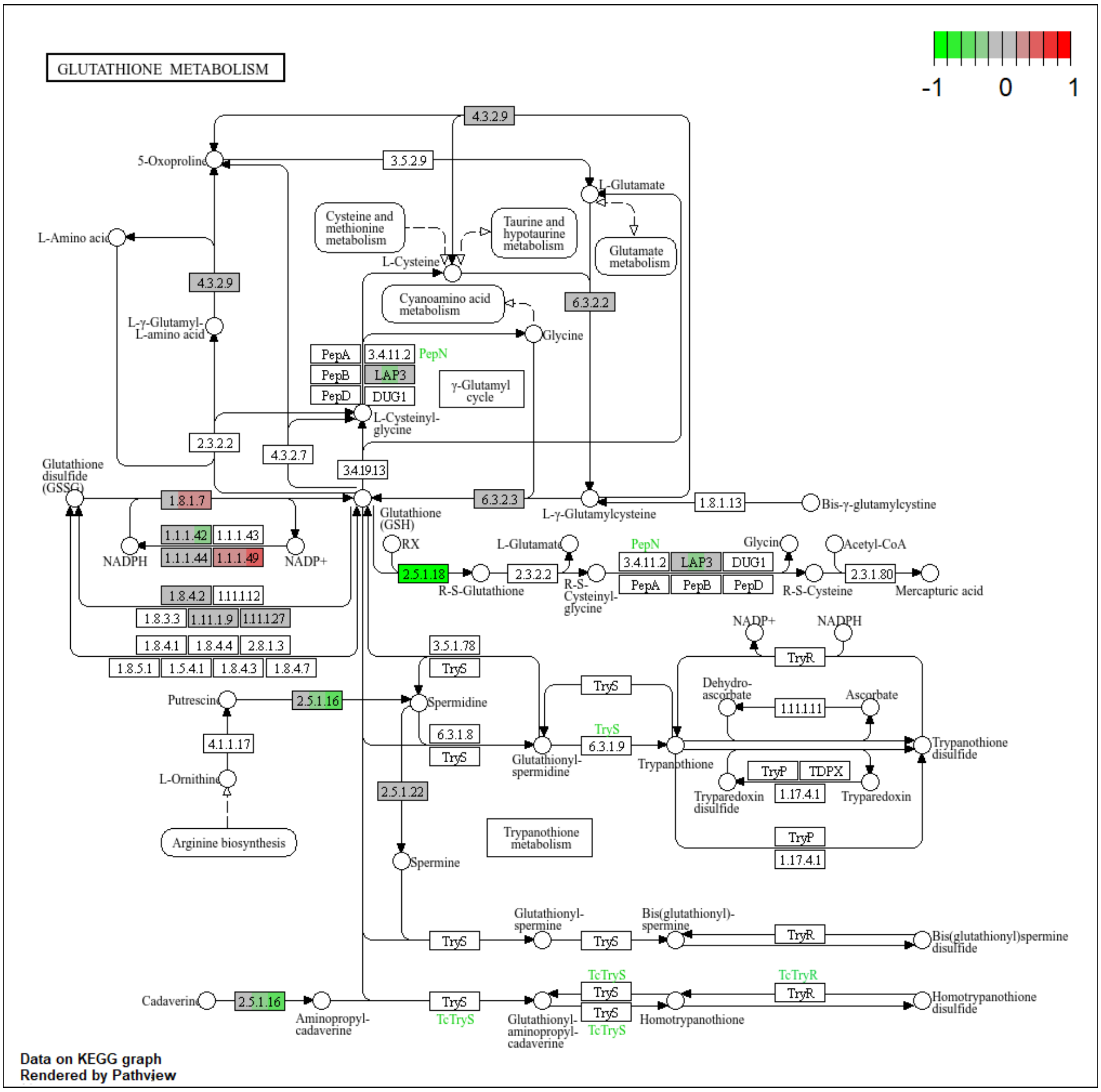

Figure S8. The KEGG glutathione metabolism pathway for the cytoplasmatic fraction of the liver. The colour of the boxes represents the log_2 fold change in the protein abundances, represented simultaneously for all three comparisons, on the left for A vs. C, in the middle for M vs. C and on the right for AM vs. C in the corresponding box for each protein.

Figure S9

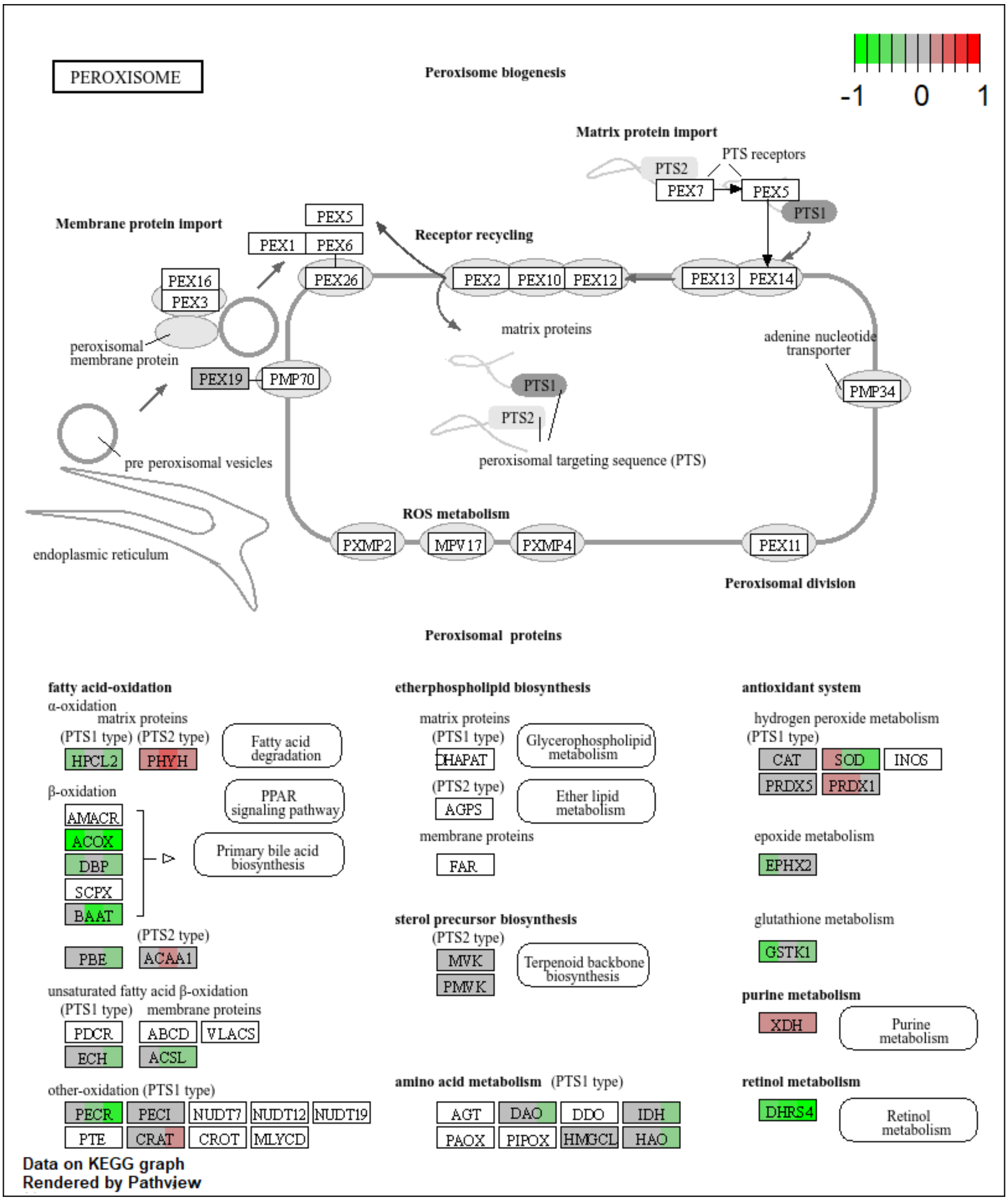

Figure S9. The KEGG peroxisome pathway for the cytoplasmatic fraction of the liver. The colour of the boxes represents the log_2 fold change in the protein abundances, represented simultaneously for all three comparisons, on the left for A vs. C, in the middle for M vs. C and on the right for AM vs. C in the corresponding box for each protein.

Figure S10

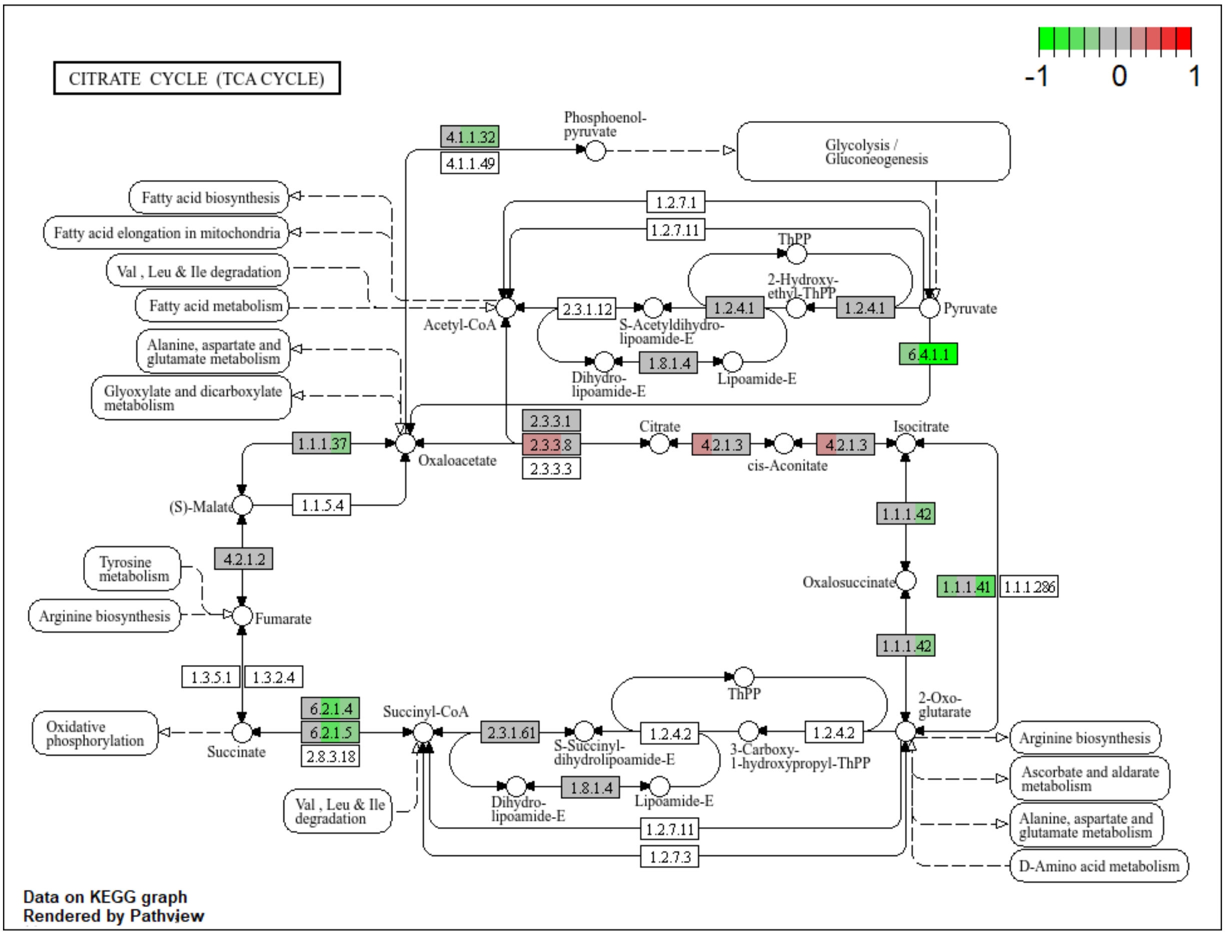

Figure S10. The KEGG TCA cycle for the cytoplasmatic fraction of the liver. The colour of the boxes represents the log_2 fold change in the protein abundances, represented simultaneously for all three comparisons, on the left for A vs. C, in the middle for M vs. C and on the right for AM vs. C in the corresponding box for each protein.

Figure S11

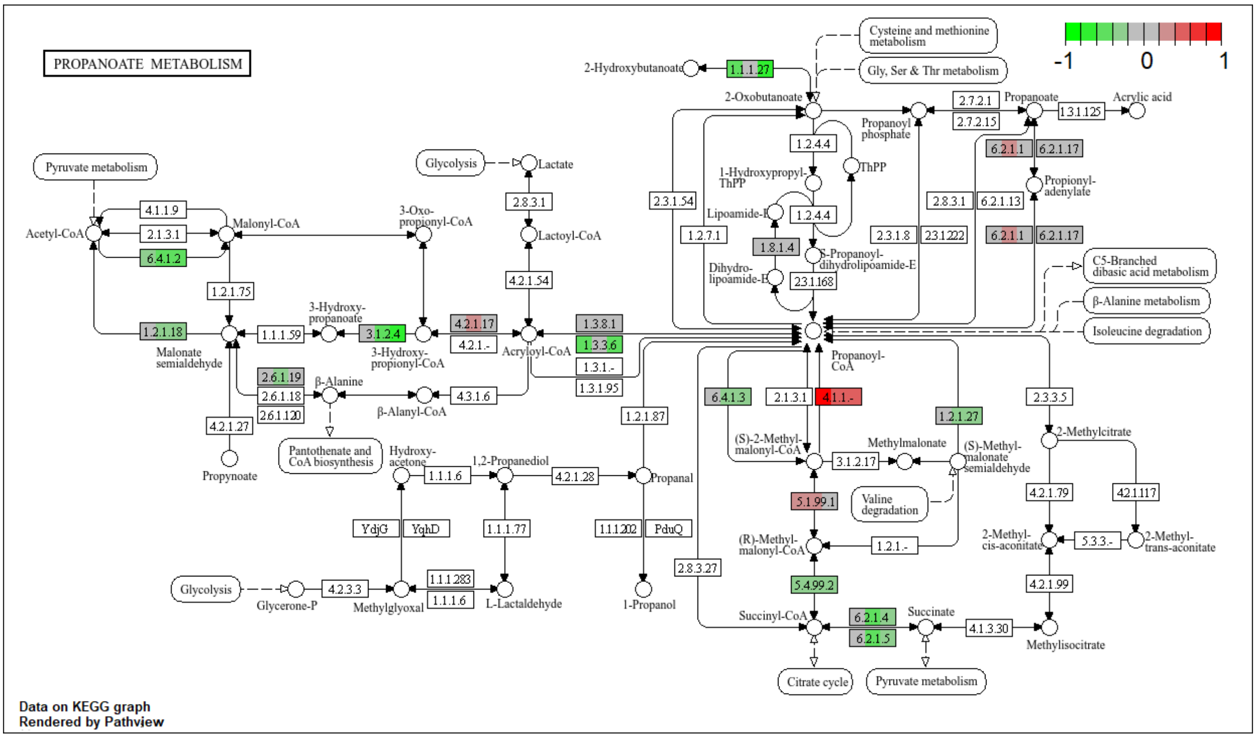

Figure S11. The KEGG propanoate metabolism for the cytoplasmatic fraction of the liver. The colour of the boxes represents the log_2 fold change in the protein abundances, represented simultaneously for all three comparisons, on the left for A vs. C, in the middle for M vs. C and on the right for AM vs. C in the corresponding box for each protein.

Figure S12

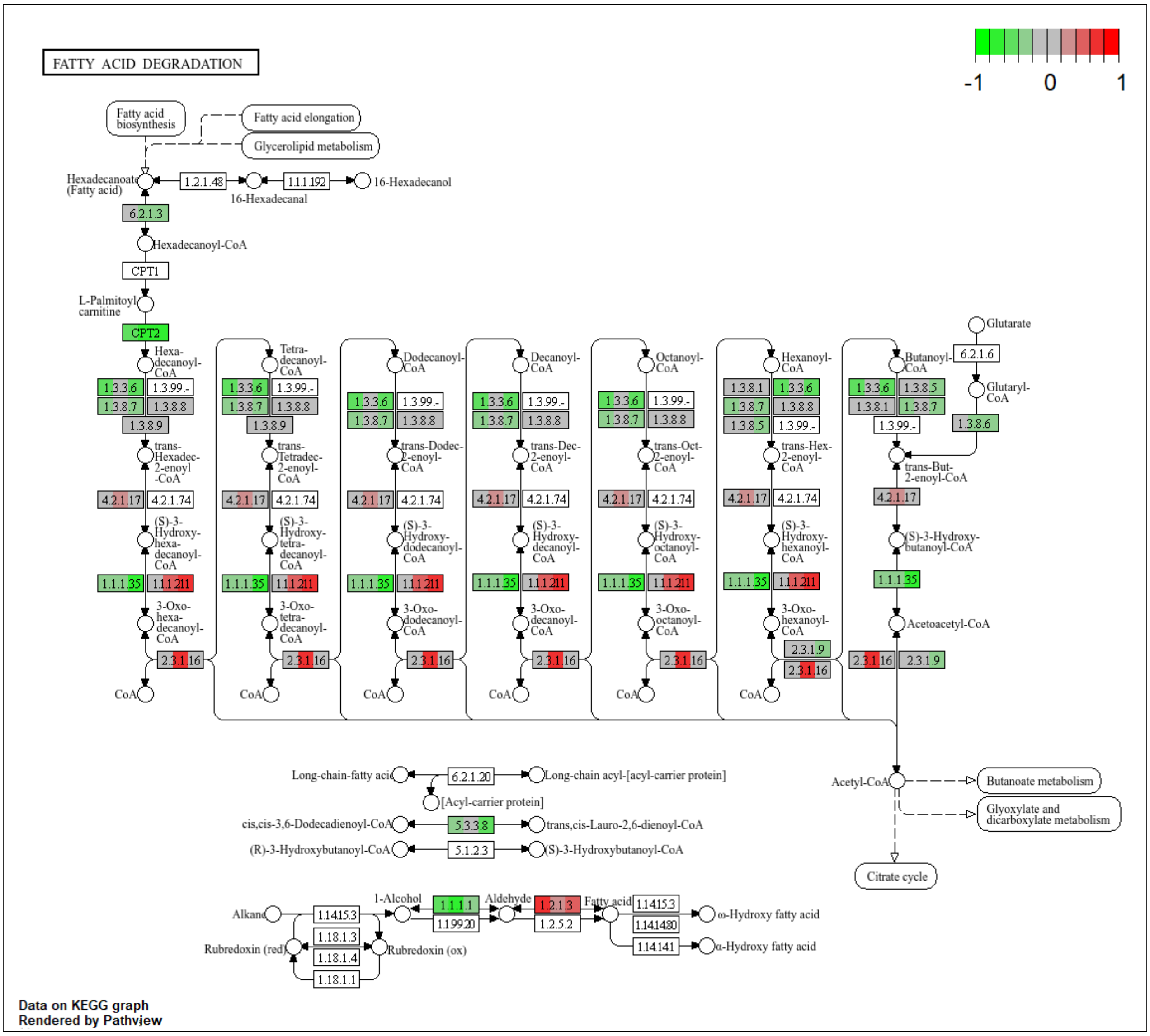

Figure S12. The KEGG fatty acid degradation for the cytoplasmatic fraction of the liver. The colour of the boxes represents the $\log_2$ fold change in the protein abundances, represented simultaneously for all three comparisons, on the left for A vs. C, in the middle for M vs. C and on the right for AM vs. C in the corresponding box for each protein.

Figure S13

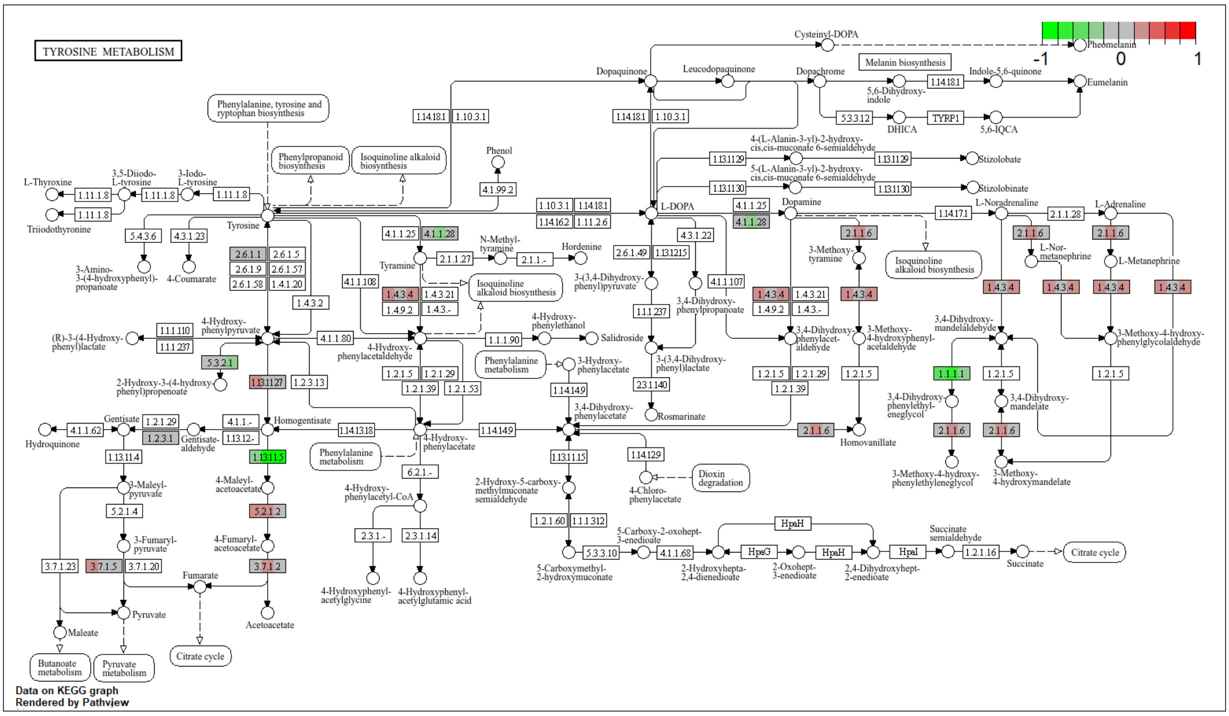

Figure S13. The KEGG tyrosine metabolism for the cytoplasmatic fraction of the liver. The colour of the boxes represents the log_2 fold change in the protein abundances, represented simultaneously for all three comparisons, on the left for A vs. C, in the middle for M vs. C and on the right for AM vs. C in the corresponding box for each protein.

Figure S14

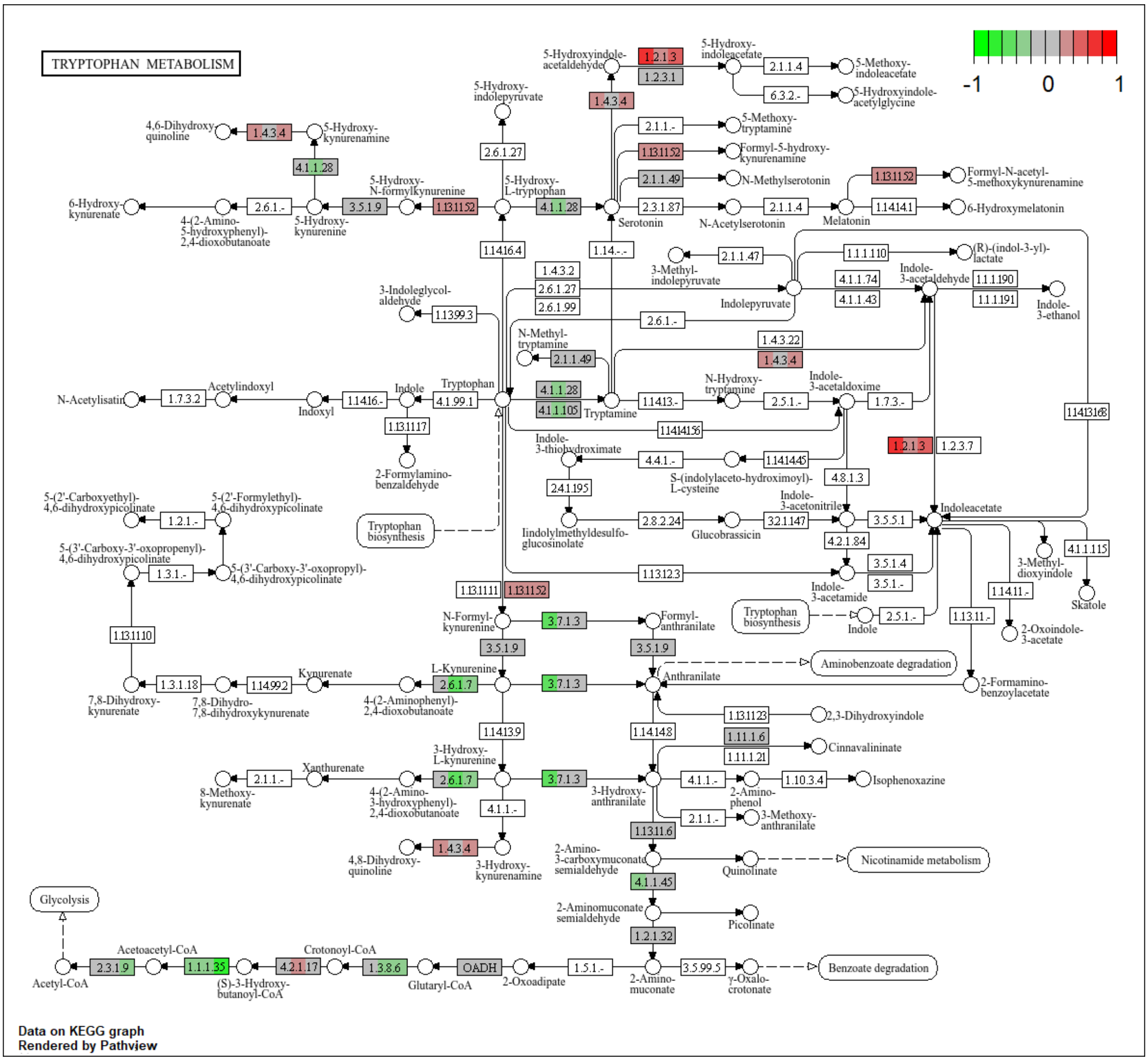

Figure S14. The KEGG tryptophan metabolism for the cytoplasmatic fraction of the liver. The colour of the boxes represents the log_2 fold change in the protein abundances, represented simultaneously for all three comparisons, on the left for A vs. C, in the middle for M vs. C and on the right for AM vs. C in the corresponding box for each protein.

Figure S15

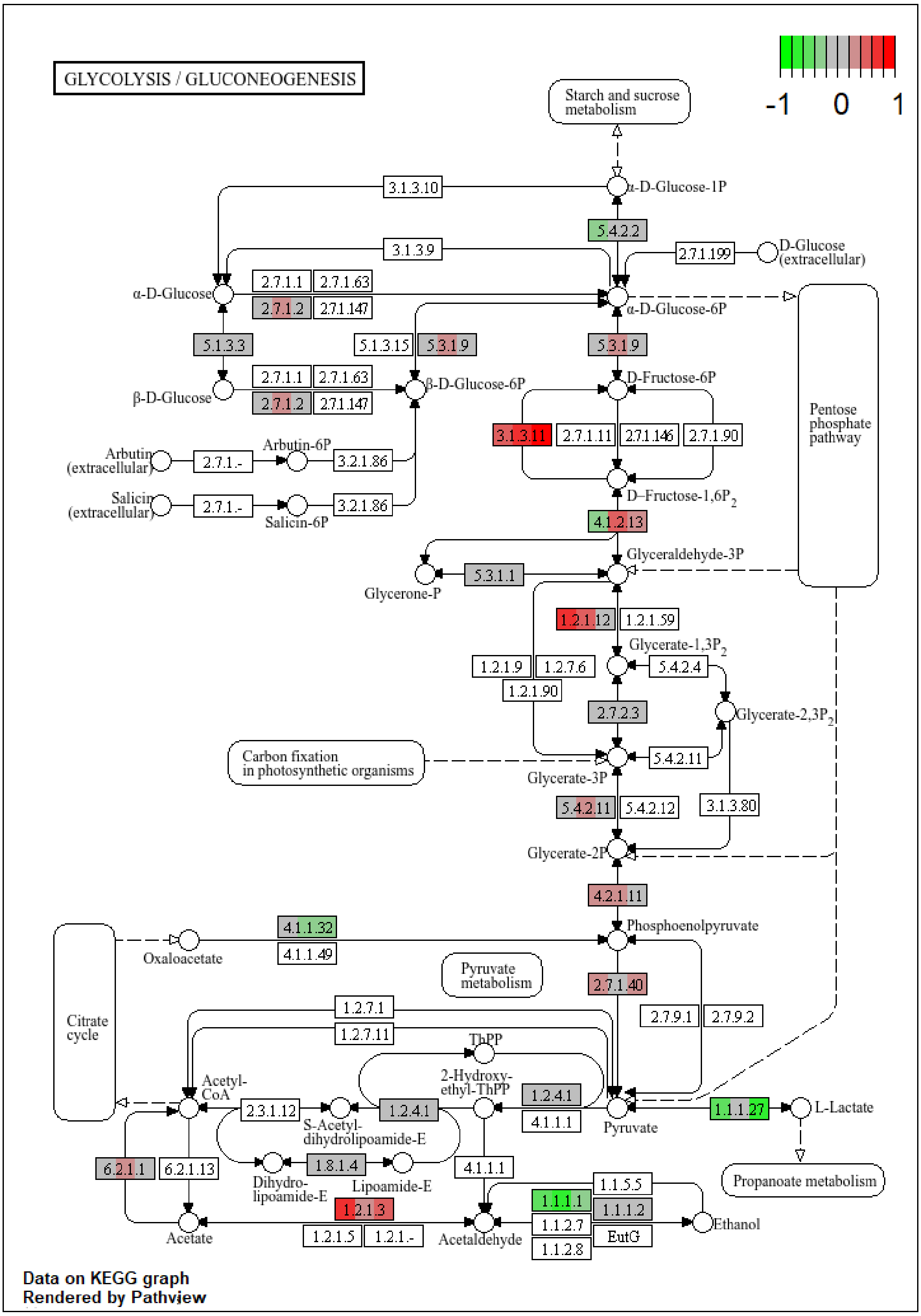

Figure S15. The KEGG glycolysis /gluconeogenesis pathway for the cytoplasmatic fraction of the liver. The colour of the boxes represents the log_2 fold change in the protein abundances, represented simultaneously for all three comparisons, on the left for A vs. C, in the middle for M vs. C and on the right for AM vs. C in the corresponding box for each protein.

Figure S16

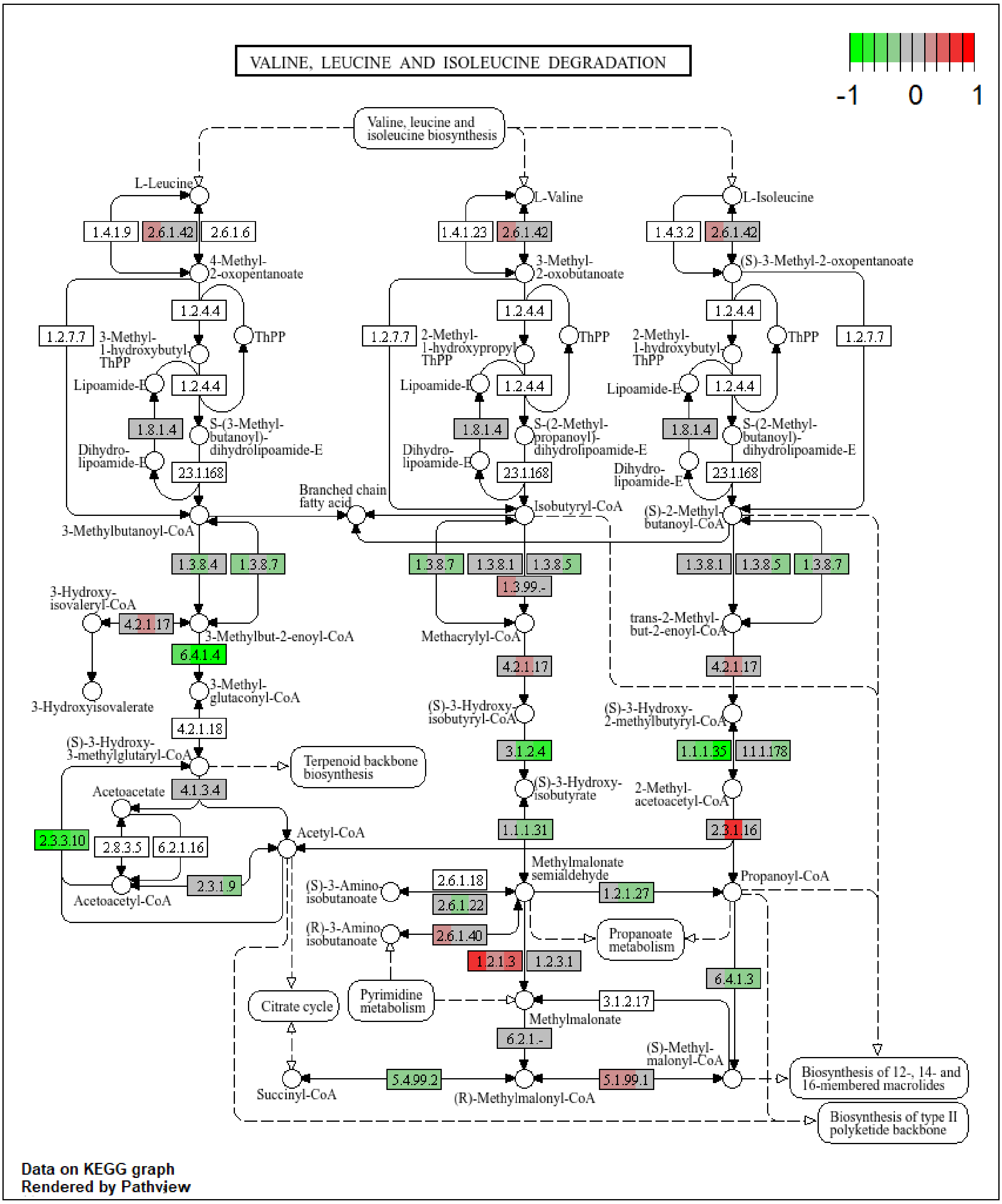

Figure S16. The KEGG valine, leucine and isoleucine degradation pathway for the cytoplasmatic fraction of the liver. The colour of the boxes represents the $\log_2$ fold change in the protein abundances, represented simultaneously for all three comparisons, on the left for A vs. C, in the middle for M vs. C and on the right for AM vs. C in the corresponding box for each protein.

Figure S17

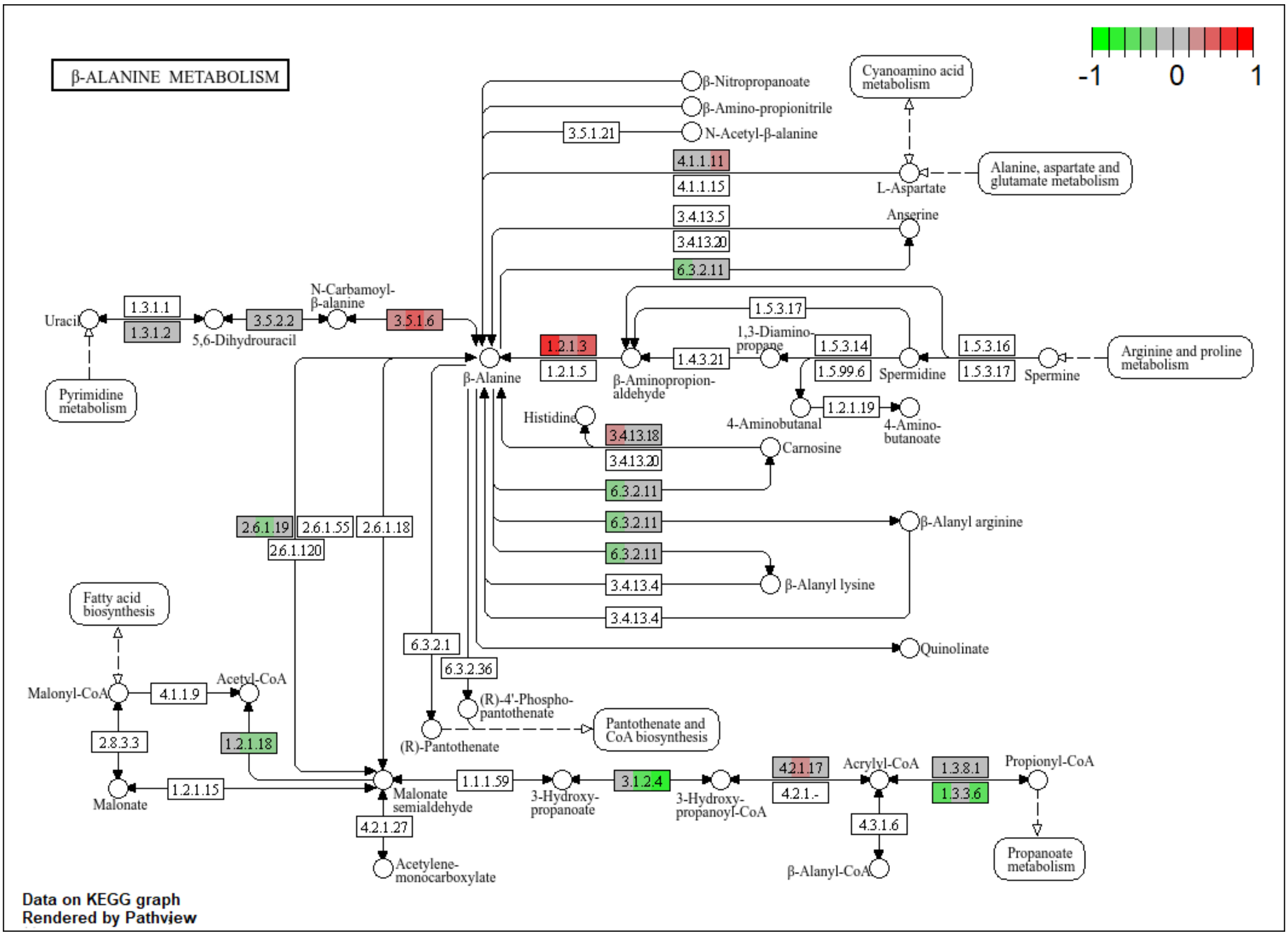

Figure S17. The KEGG beta-alanine pathway for the cytoplasmatic fraction of the liver. The colour of the boxes represents the log_2 fold change in the protein abundances, represented simultaneously for all three comparisons, on the left for A vs. C, in the middle for M vs. C and on the right for AM vs. C in the corresponding box for each protein.

Figure S18

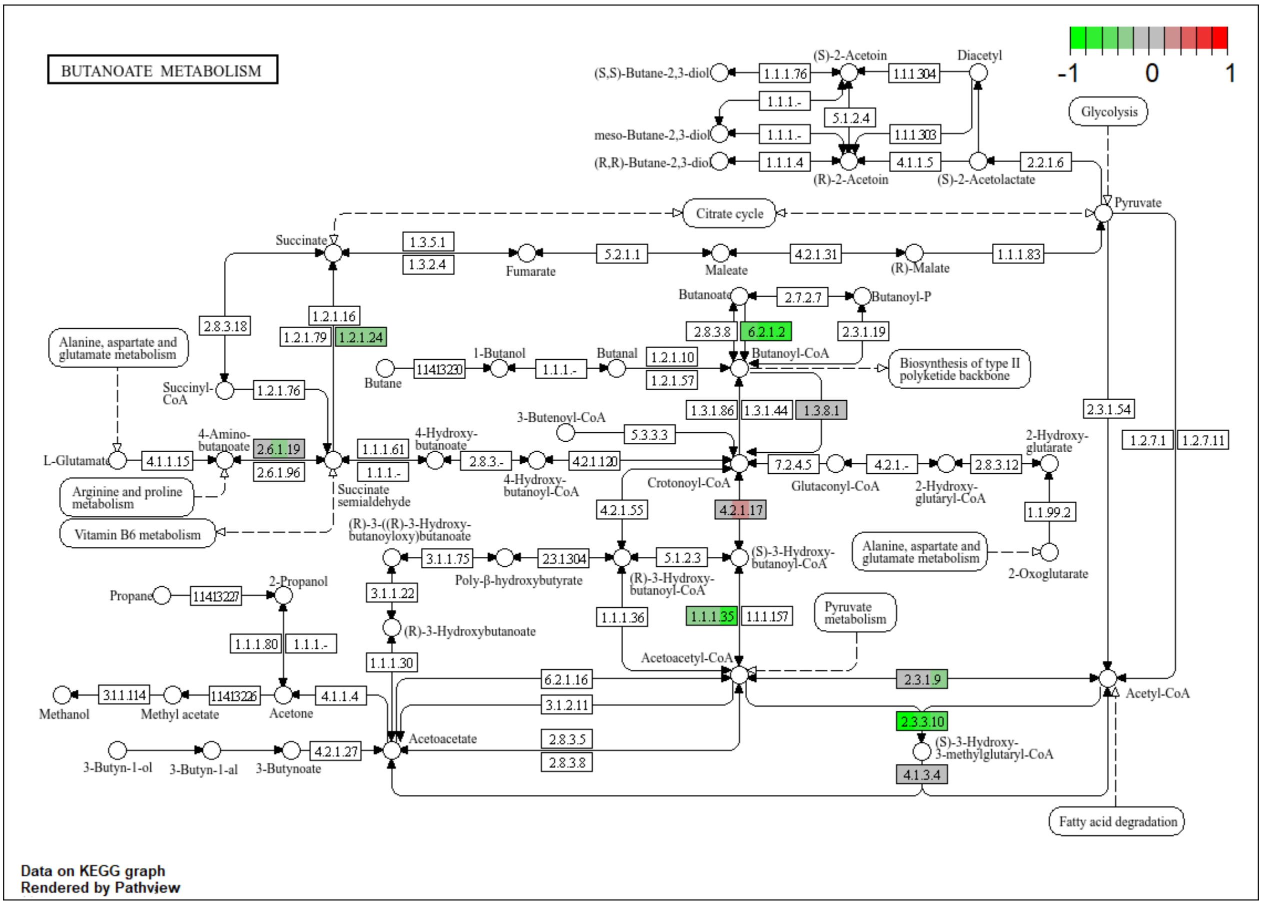

Figure S18. The KEGG butanoate metabolism for the cytoplasmatic fraction of the liver. The colour of the boxes represents the $\log_2$ fold change in the protein abundances, represented simultaneously for all three comparisons, on the left for A vs. C, in the middle for M vs. C and on the right for AM vs. C in the corresponding box for each protein.

Figure S19

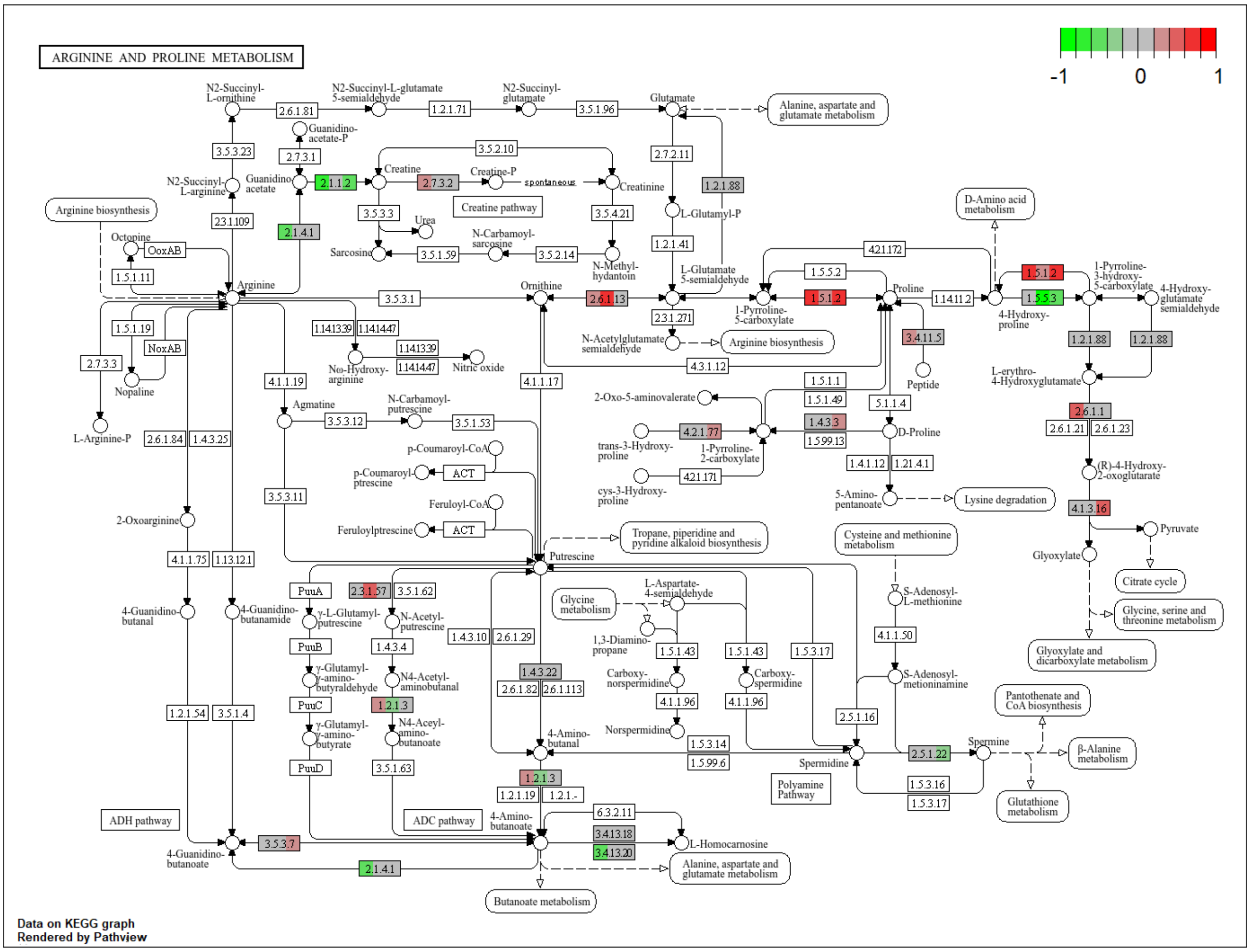

Figure S19. The KEGG arginine and proline metabolism for the cytoplasmatic fraction of the kidney. The colour of the boxes represents the log_2 fold change in the protein abundances, represented simultaneously for all three comparisons, on the left for A vs. C, in the middle for M vs. C and on the right for AM vs. C in the corresponding box for each protein.

Figure S20

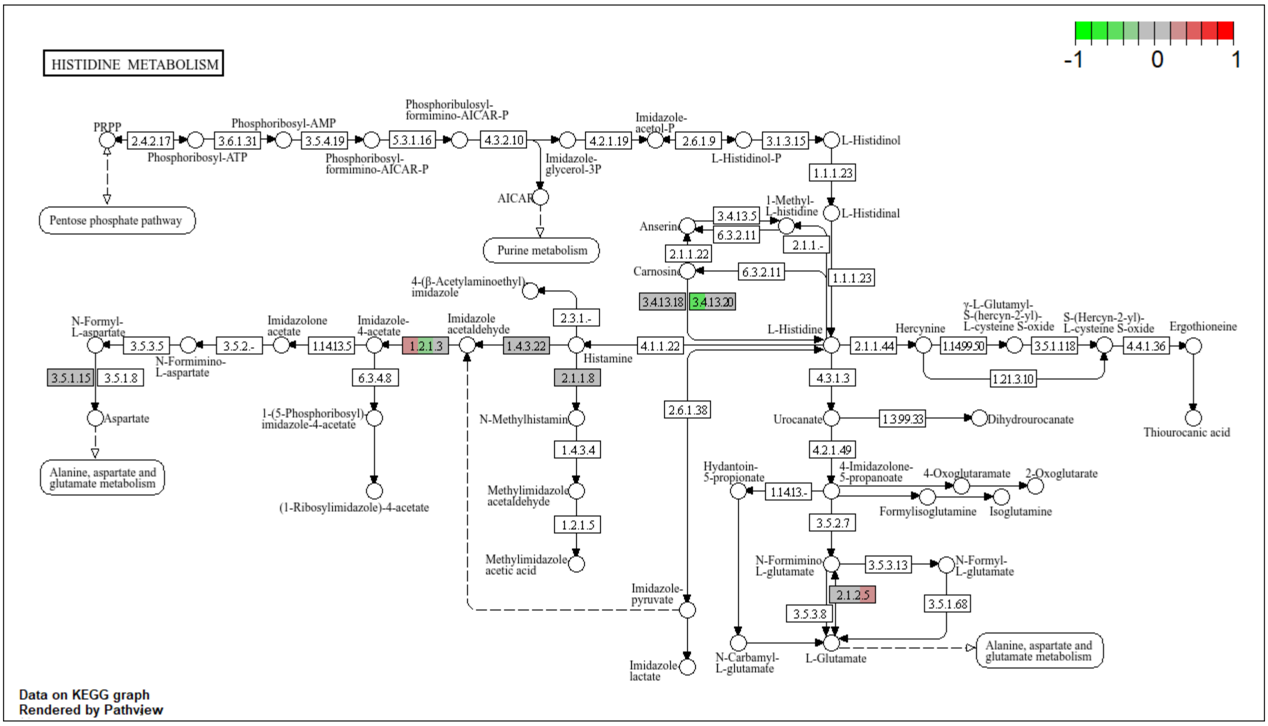

Figure S20. The KEGG histidine metabolism for the cytoplasmatic fraction of the kidney. The colour of the boxes represents the $\log_2$ fold change in the protein abundances, represented simultaneously for all three comparisons, on the left for A vs. C, in the middle for M vs. C and on the right for AM vs. C in the corresponding box for each protein.

Figure S21

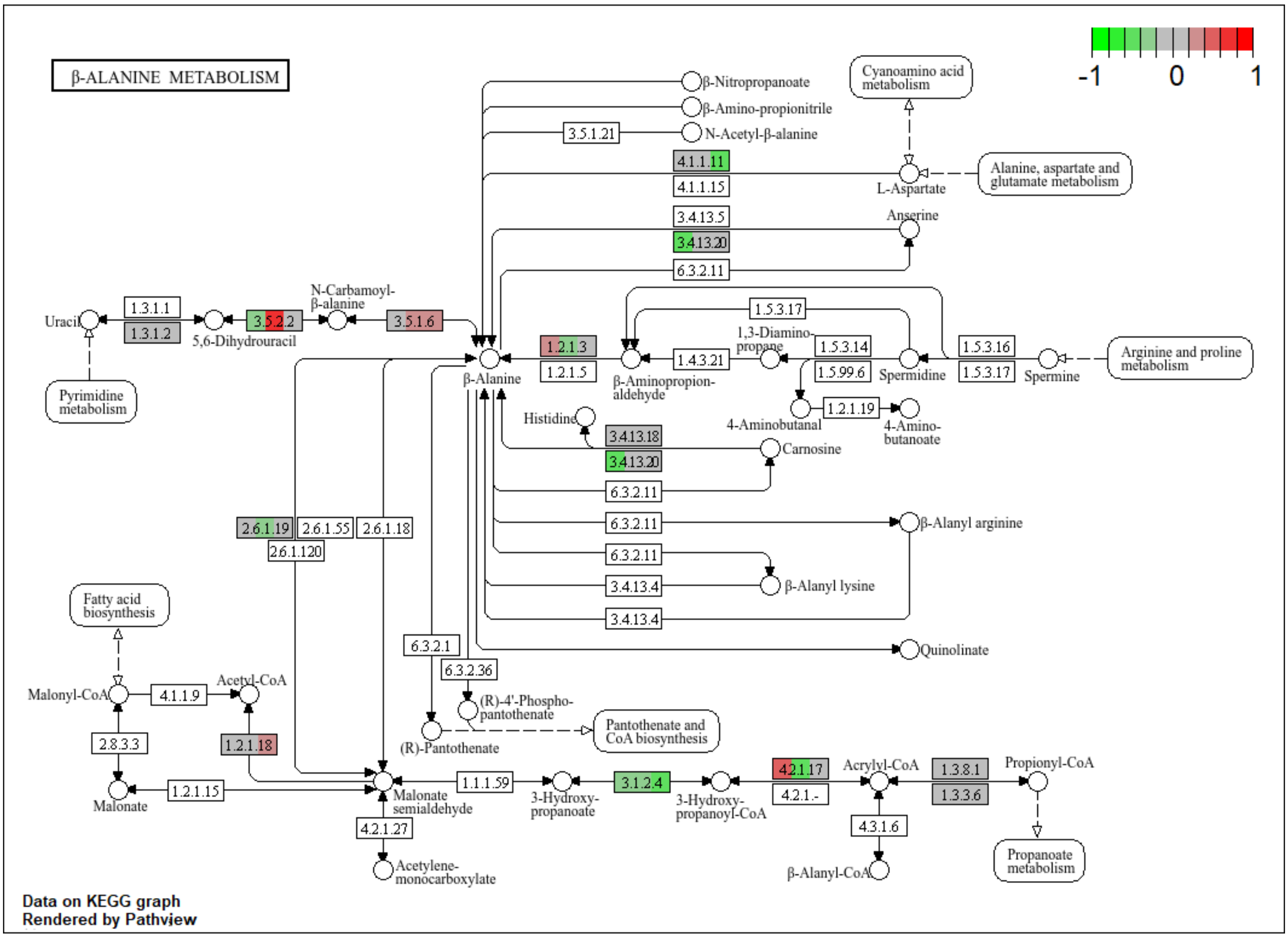

Figure S21. The KEGG beta-alanine metabolism for the cytoplasmatic fraction of the kidney. The colour of the boxes represents the log_2 fold change in the protein abundances, represented simultaneously for all three comparisons, on the left for A vs. C, in the middle for M vs. C and on the right for AM vs. C in the corresponding box for each protein.

Figure S22

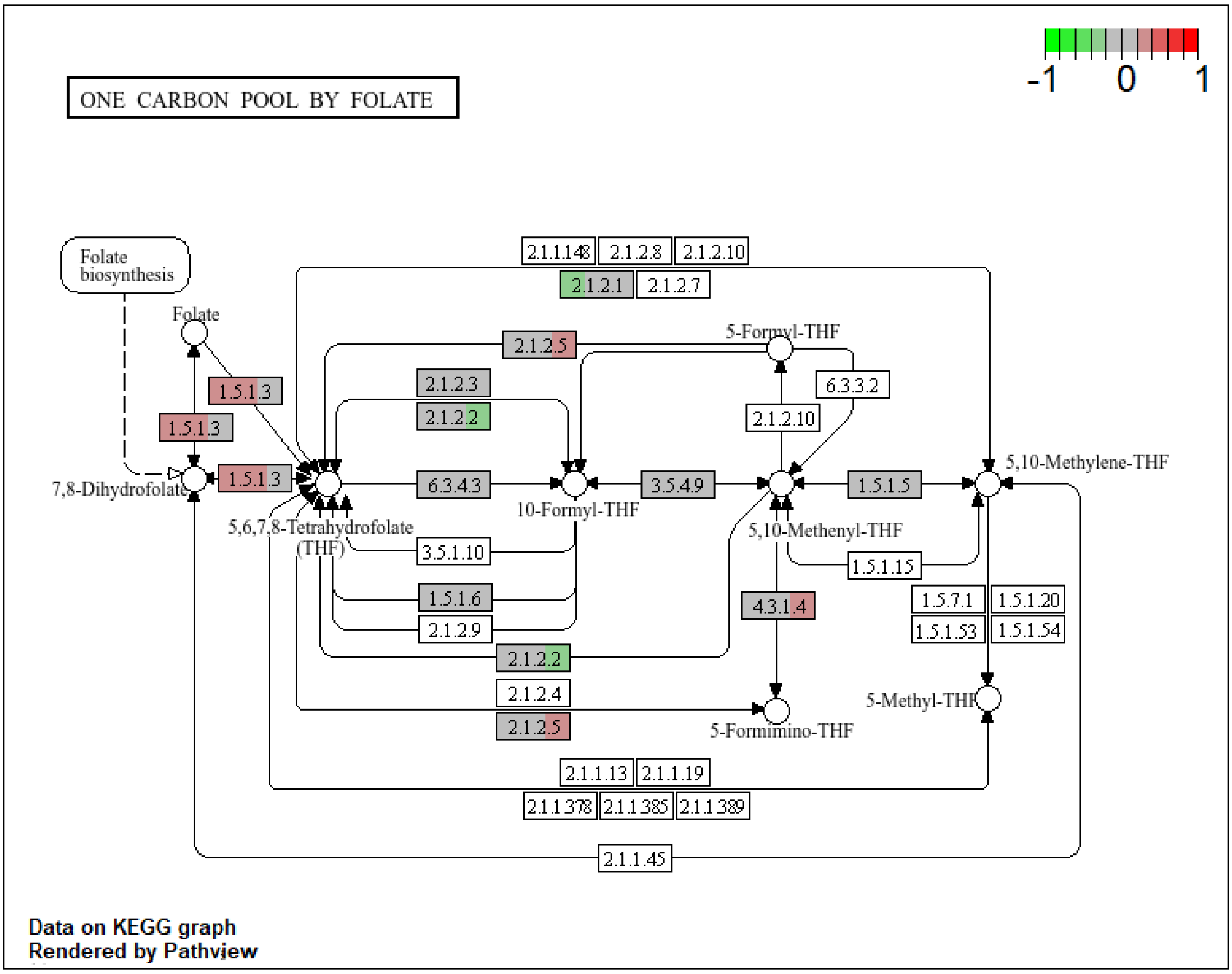

Figure S22. The KEGG one carbon pool by folate pathway for the cytoplasmatic fraction of the kidney. The colour of the boxes represents the log_2 fold change in the protein abundances, represented simultaneously for all three comparisons, on the left for A vs. C, in the middle for M vs. C and on the right for AM vs. C in the corresponding box for each protein.

Figure S23

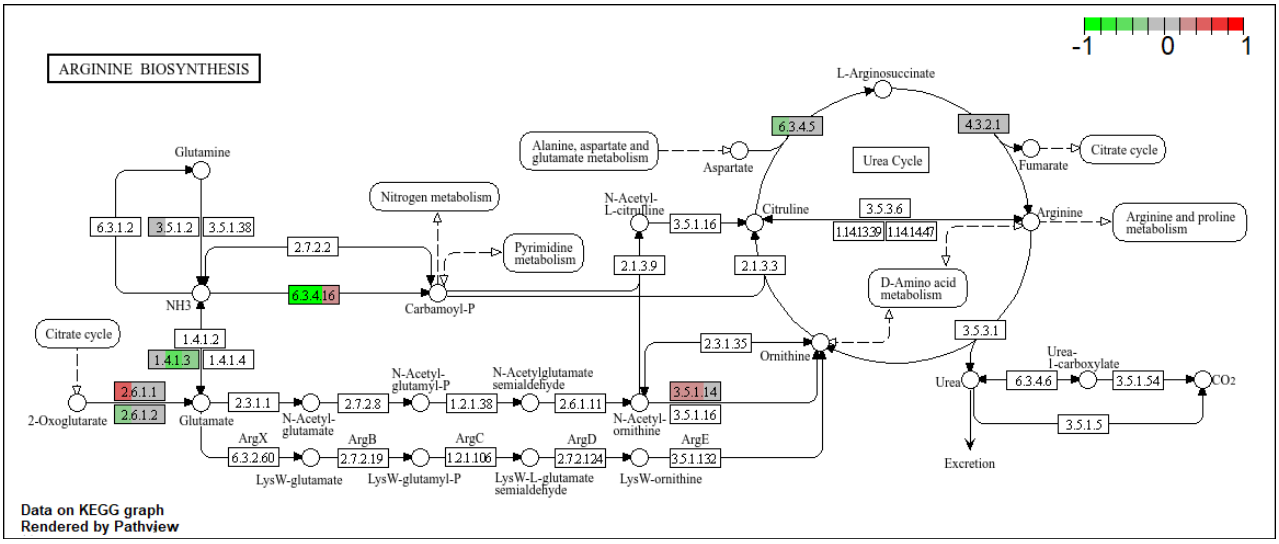

Figure S23. The KEGG arginine biosynthesis pathway for the cytoplasmatic fraction of the kidney. The colour of the boxes represents the log_2 fold change in the protein abundances, represented simultaneously for all three comparisons, on the left for A vs. C, in the middle for M vs. C and on the right for AM vs. C in the corresponding box for each protein.

Figure S24

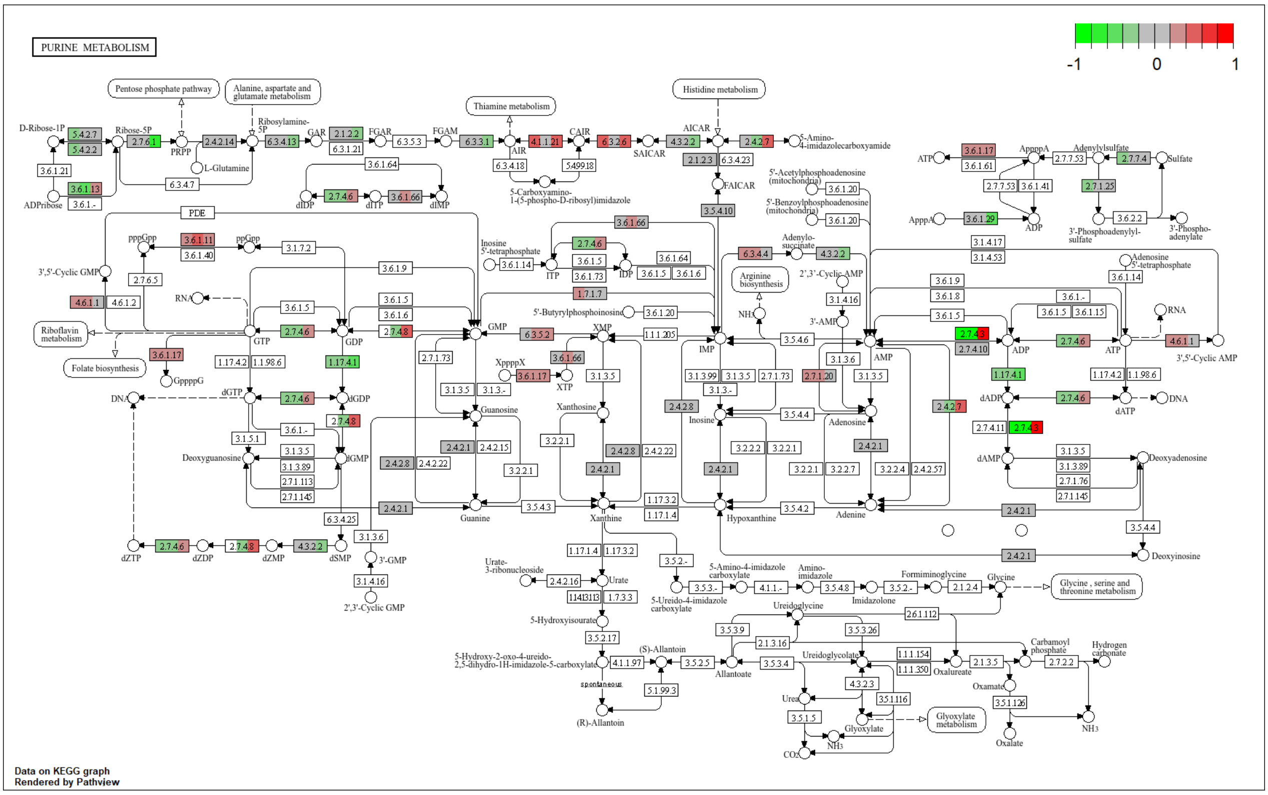

Figure S24. The KEGG purine metabolism for the cytoplasmatic fraction of the kidney. The colour of the boxes represents the log_2 fold change in the protein abundances, represented simultaneously for all three comparisons, on the left for A vs. C, in the middle for M vs. C and on the right for AM vs. C in the corresponding box for each protein.

Figure S25

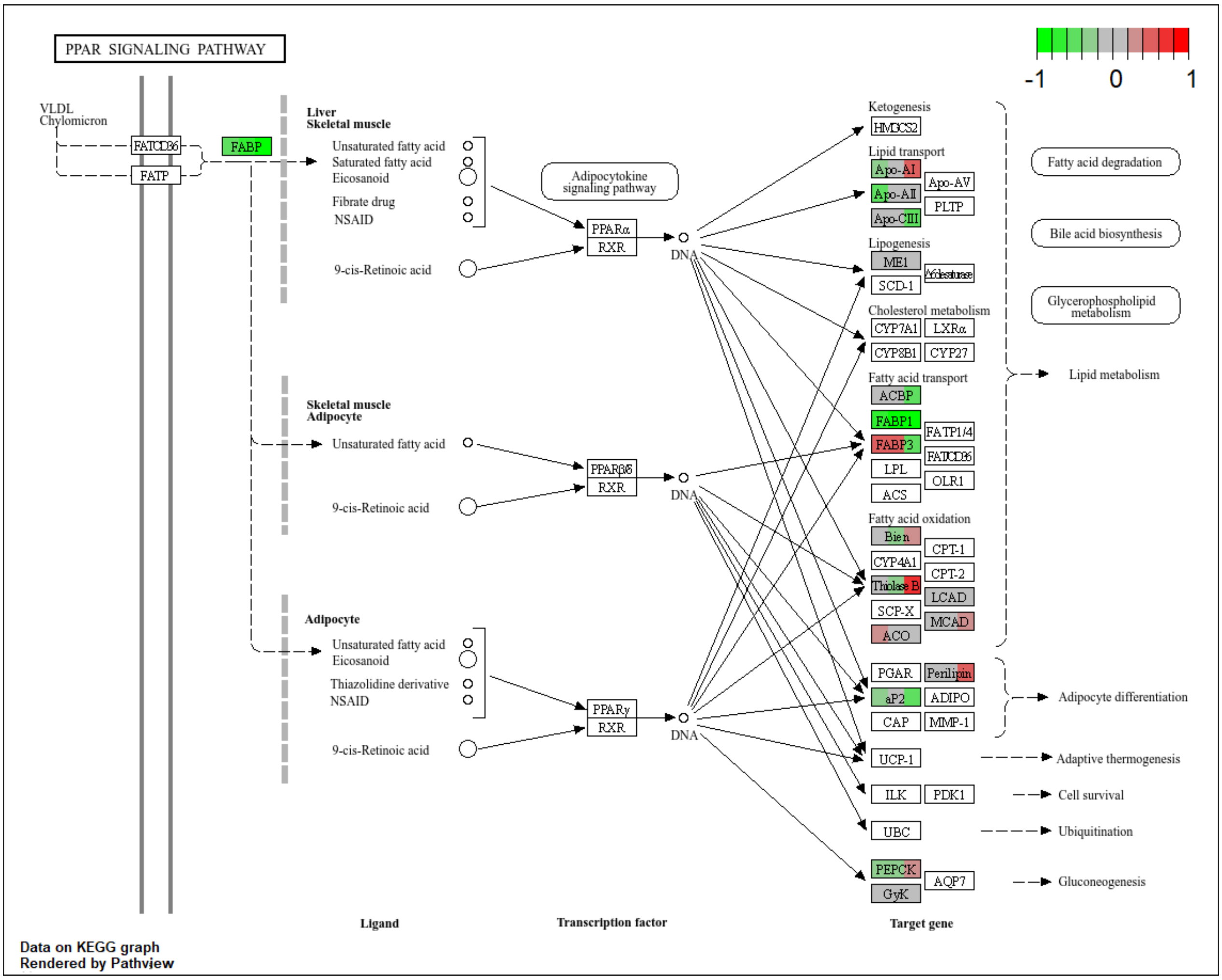

Figure S25. The KEGG PPAR signalling pathway for the cytoplasmatic fraction of the kidney. The colour of the boxes represents the log$_2$ fold change in the protein abundances, represented simultaneously for all three comparisons, on the left for A vs. C, in the middle for M vs. C and on the right for AM vs. C in the corresponding box for each protein.

Figure S26

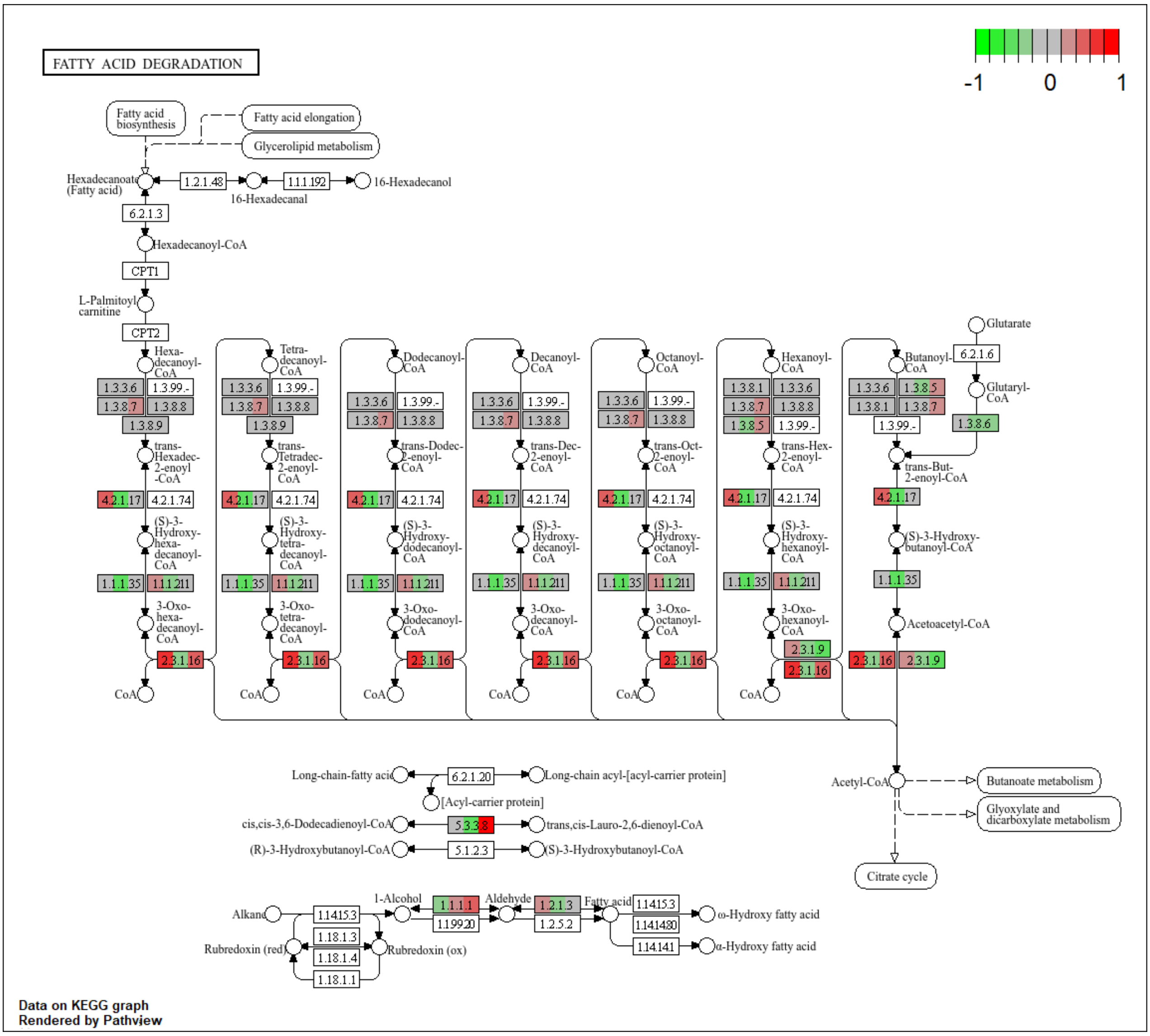

Figure S26. The KEGG fatty acid degradation pathway for the cytoplasmatic fraction of the kidney. The colour of the boxes represents the log_2 fold change in the protein abundances, represented simultaneously for all three comparisons, on the left for A vs. C, in the middle for M vs. C and on the right for AM vs. C in the corresponding box for each protein.

Figure S27

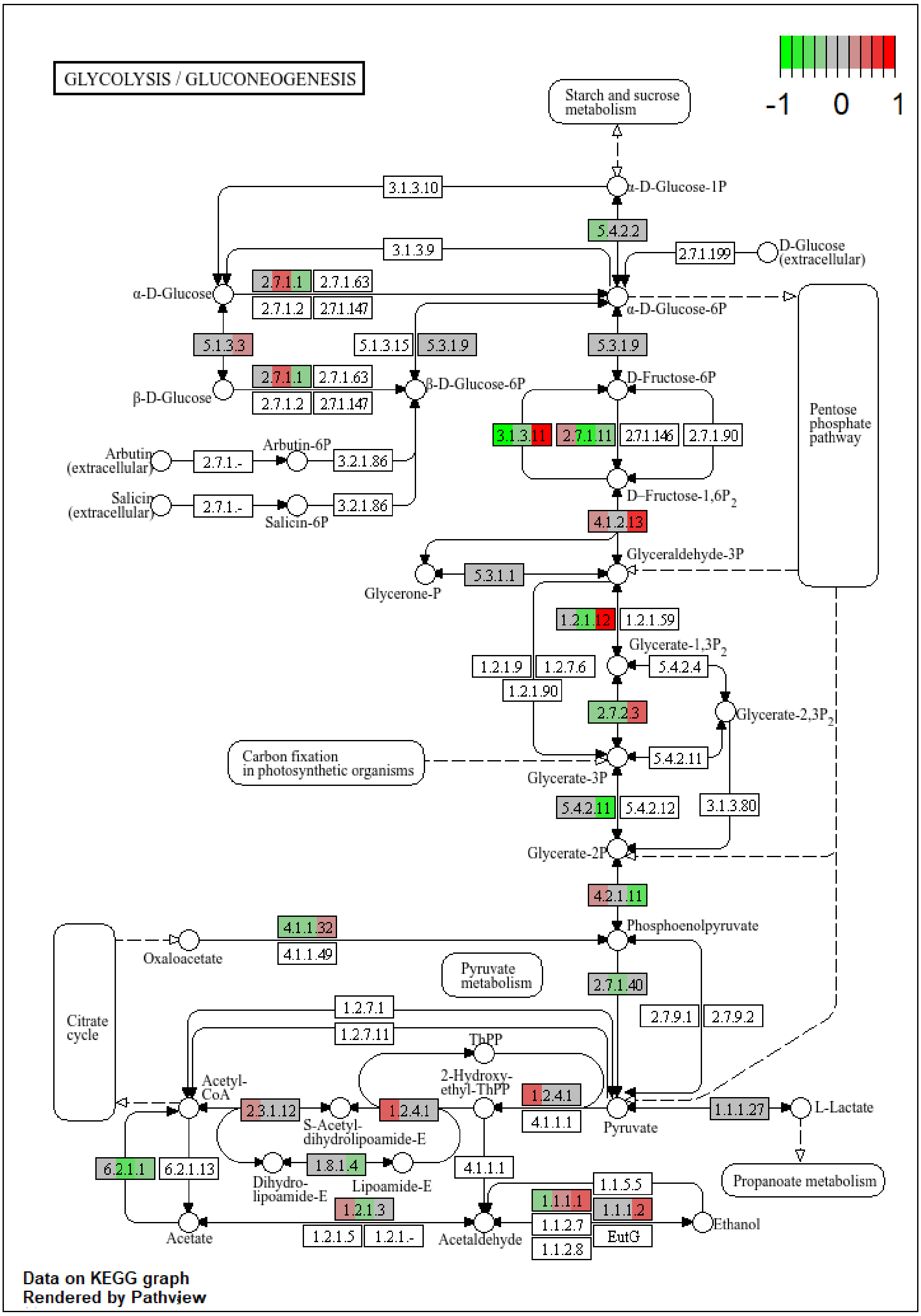

Figure S27. The KEGG glycolysis /gluconeogenesis pathway for the cytoplasmatic fraction of the kidney. The colour of the boxes represents the $\log_2$ fold change in the protein abundances, represented simultaneously for all three comparisons, on the left for A vs. C, in the middle for M vs. C and on the right for AM vs. C in the corresponding box for each protein.

Figure S28

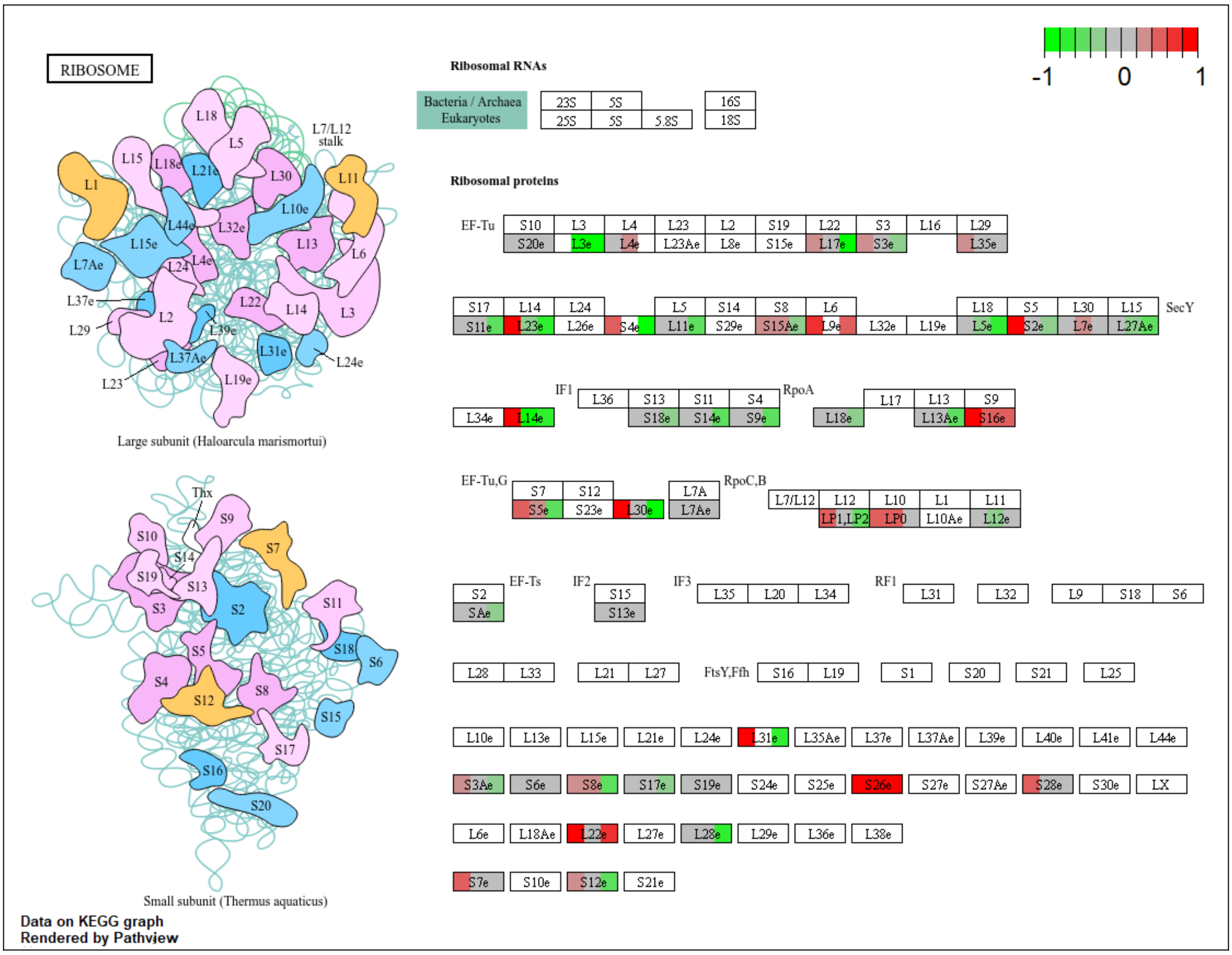

Figure S28. The KEGG ribosome pathway for the cytoplasmatic fraction of the kidney. The colour of the boxes represents the log_2 fold change in the protein abundances, represented simultaneously for all three comparisons, on the left for A vs. C, in the middle for M vs. C and on the right for AM vs. C in the corresponding box for each protein.

Figure S29

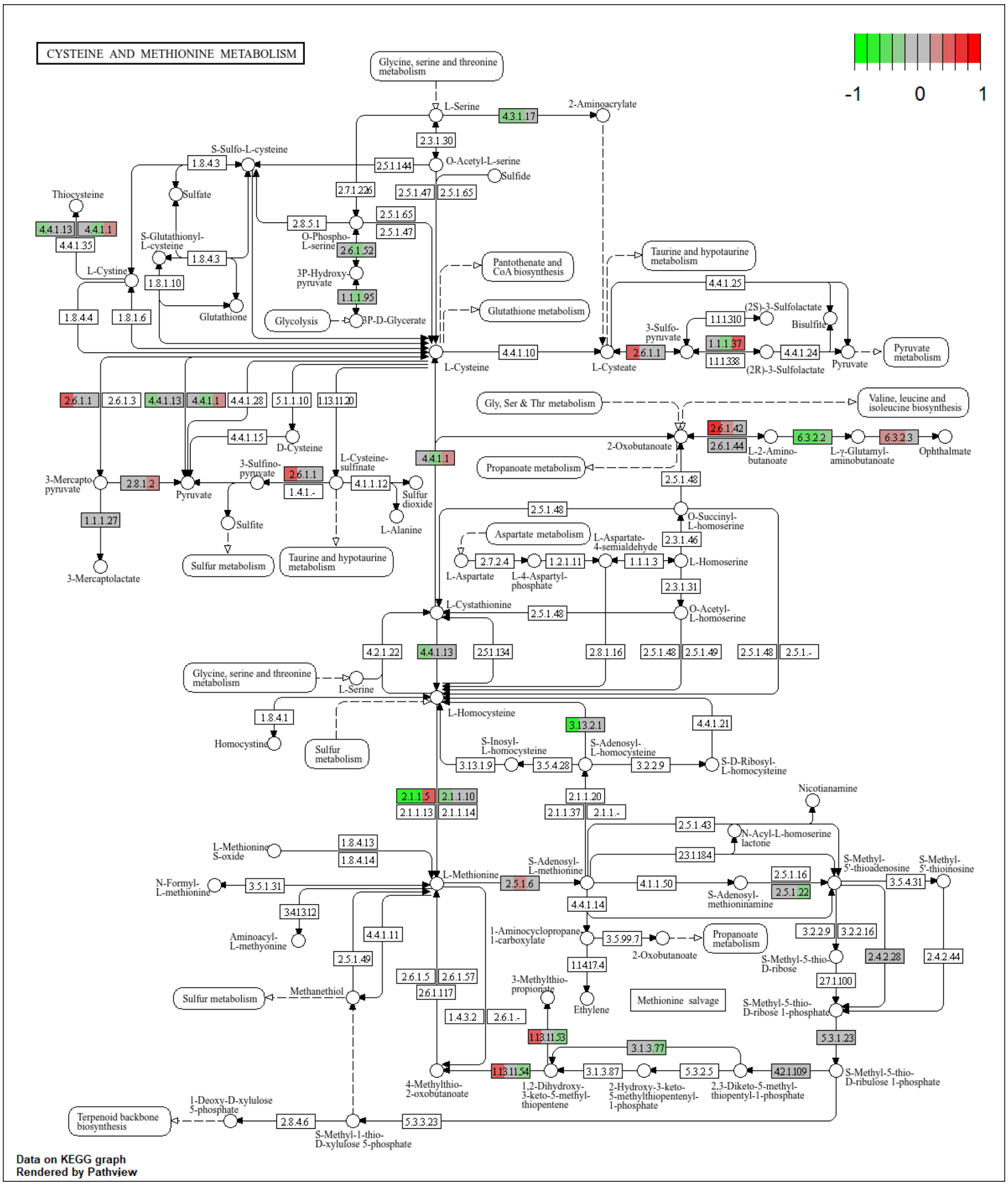

Figure S29. The KEGG cysteine and methionine metabolism for the cytoplasmatic fraction of the kidney. The colour of the boxes represents the log_2 fold change in the protein abundances, represented simultaneously for all three comparisons, on the left for A vs. C, in the middle for M vs. C and on the right for AM vs. C in the corresponding box for each protein.

Figure S30

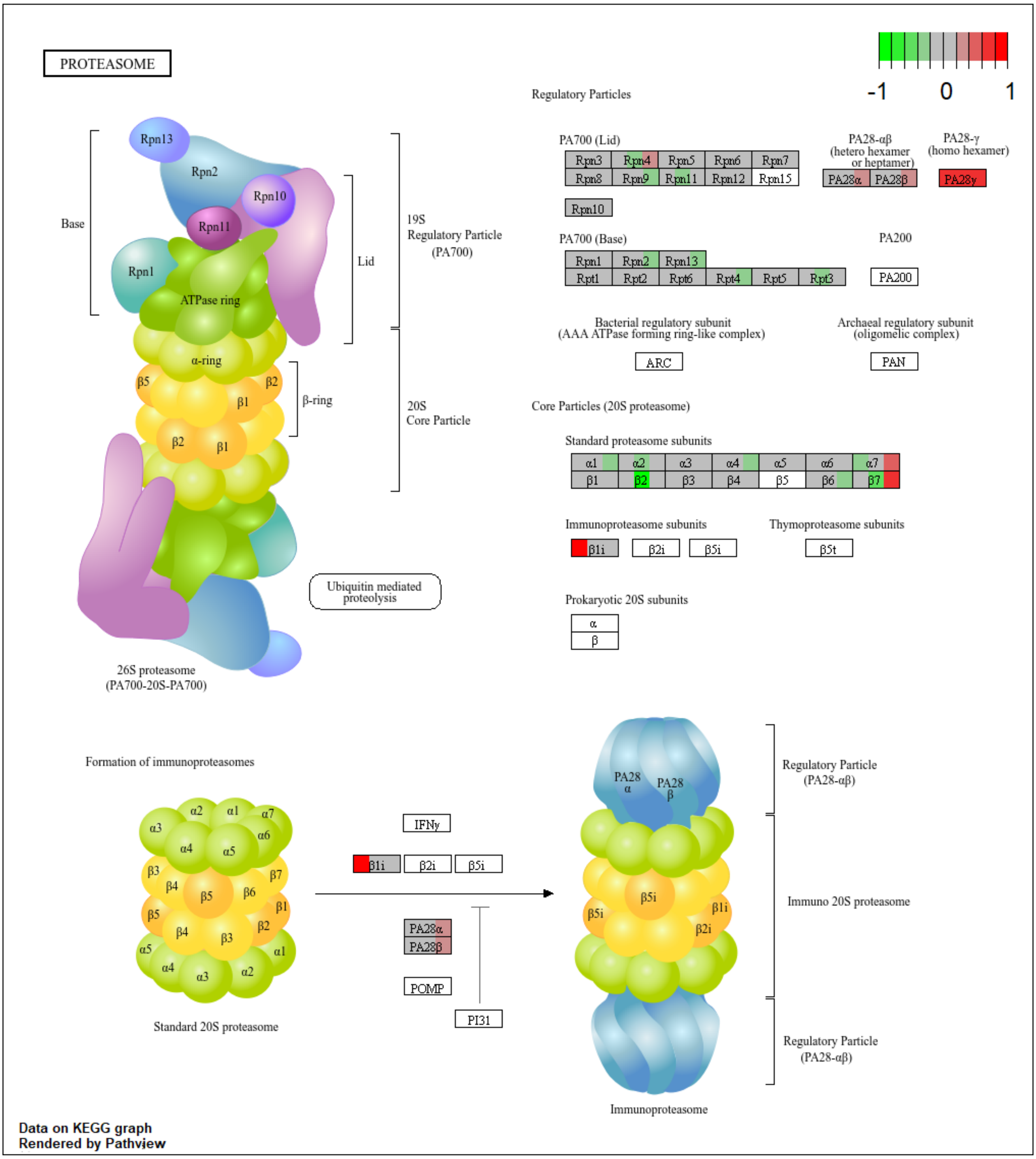

Figure S30. The KEGG proteasome pathway for the cytoplasmatic fraction of the kidney. The colour of the boxes represents the log_2 fold change in the protein abundances, represented simultaneously for all three comparisons, on the left for A vs. C, in the middle for M vs. C and on the right for AM vs. C in the corresponding box for each protein.

Figure S31

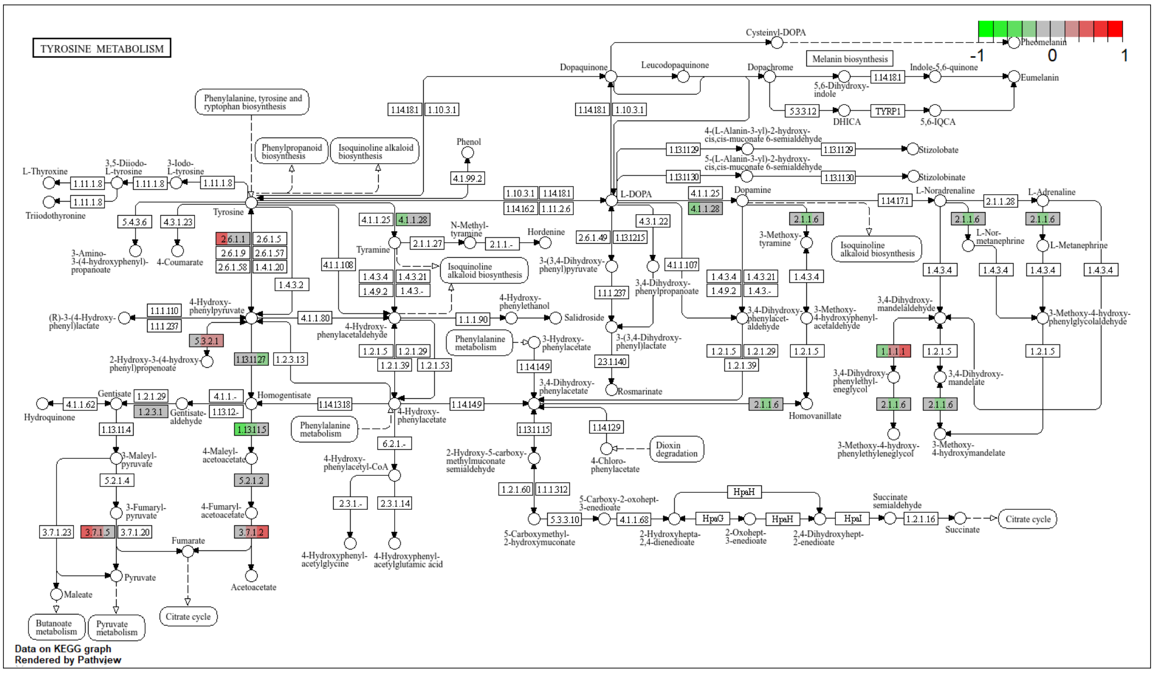

Figure S31. The KEGG tyrosine metabolism pathway for the cytoplasmatic fraction of the kidney. The colour of the boxes represents the log_2 fold change in the protein abundances, represented simultaneously for all three comparisons, on the left for A vs. C, in the middle for M vs. C and on the right for AM vs. C in the corresponding box for each protein.

Figure S32

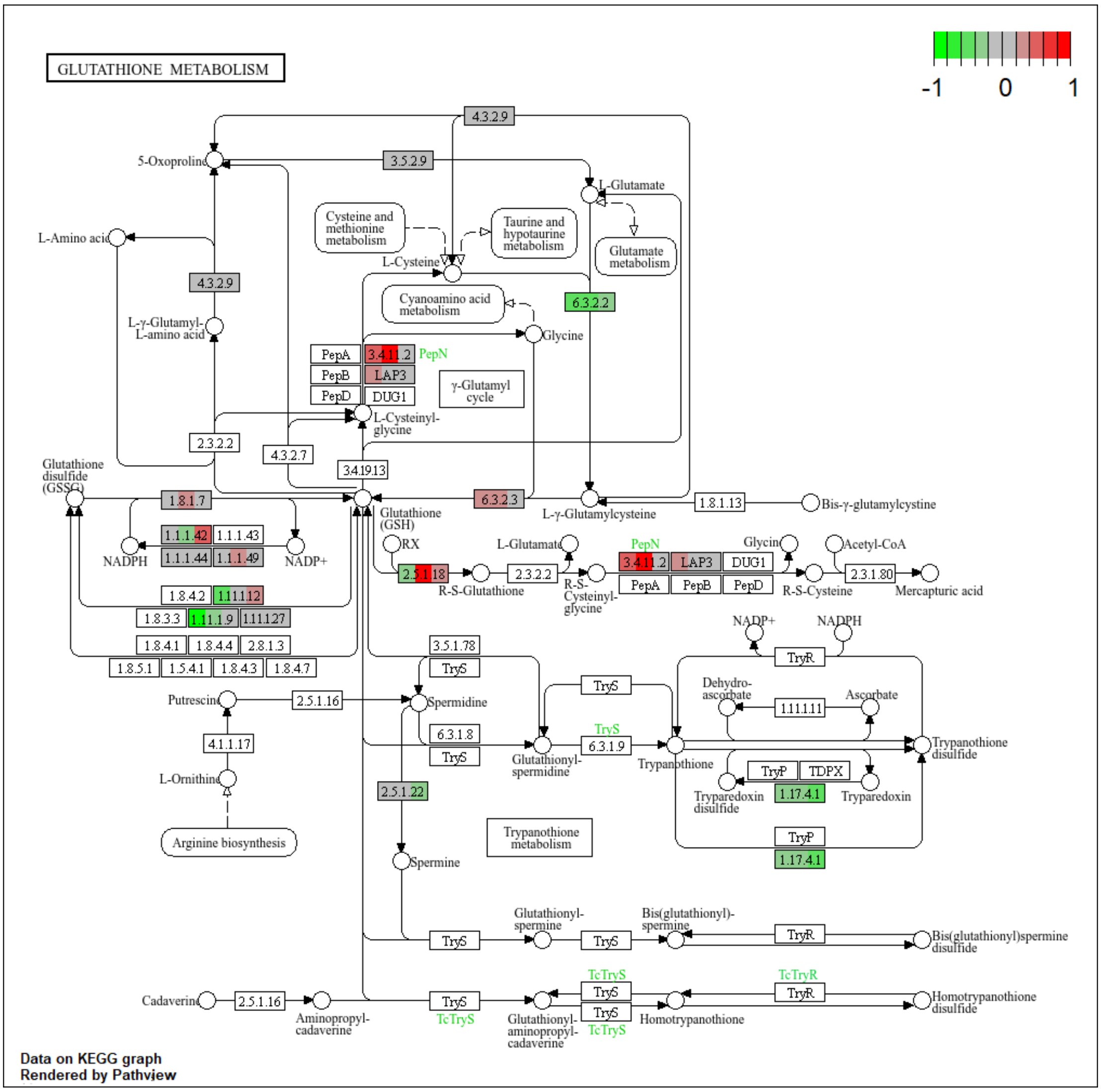

Figure S32. The KEGG glutahione metabolism pathway for the cytoplasmatic fraction of the kidney. The colour of the boxes represents the $\log_2$ fold change in the protein abundances, represented simultaneously for all three comparisons, on the left for A vs. C, in the middle for M vs. C and on the right for AM vs. C in the corresponding box for each protein.
